# Single-dose versus 3-day cotrimoxazole prophylaxis in transurethral resection or greenlight laser vaporisation of the prostate: study protocol for a multicentre randomised placebo controlled non-inferiority trial (CITrUS trial)

**DOI:** 10.1186/s13063-019-3237-3

**Published:** 2019-02-19

**Authors:** Benjamin Speich, Kathrin Bausch, Jan A. Roth, Lars G. Hemkens, Hannah Ewald, Deborah R. Vogt, Nicole Bruni, Stefanie Deuster, Hans-H. Seifert, Andreas F. Widmer

**Affiliations:** 1 Basel Institute for Clinical Epidemiology and Biostatistics, Department of Clinical Research, University Hospital Basel, University of Basel, Basel, Switzerland; 2 Centre for Statistics in Medicine, Nuffield Department of Orthopaedics, Rheumatology and Musculoskeletal Sciences, University of Oxford, Oxford, UK; 3 Department of Urology, University Hospital Basel, University of Basel, Basel, Switzerland; 4 Division of Infectious Diseases and 721 Hospital Epidemiology, University Hospital Basel, University of Basel, Petersgraben, 4031 Basel, Switzerland; 5 University Medical Library, University of Basel, Basel, Switzerland; 6 Clinical Trial Unit, Department of Clinical Research, University Hospital Basel, University of Basel, Basel, Switzerland; 7 Hospital Pharmacy, University Hospital Basel, Basel, Switzerland

**Keywords:** Transurethral resection of prostate, Greenlight laser vaporisation, Antibiotic prophylaxis, Urinary tract infection, Randomised controlled trial

## Abstract

**Background:**

Transurethral resection of the prostate (TURP) and Greenlight laser vaporisation (GL) of the prostate are frequently performed urological procedures. For TURP, a single-dose antimicrobial prophylaxis (AP) is recommended to reduce postoperative urinary tract infections. So far, no international recommendations for AP have been established for GL. In a survey-based study in Switzerland, Germany and Austria, urologists reported routinely extending AP primarily for 3 days after both interventions. We therefore aim to determine whether single-dose AP with cotrimoxazole is non-inferior to 3-day AP with cotrimoxazole in patients undergoing TURP or GL of the prostate.

**Methods/design:**

We will conduct an investigator-initiated, multicentre, randomised controlled trial. We plan to assess the non-inferiority of single-dose AP compared to 3-day AP. The primary outcome is the occurrence of clinically diagnosed symptomatic urinary tract infections which are treated with antimicrobial agents within 30 days after randomisation. The vast majority of collected outcomes will be assessed from routinely collected data. The sample size was estimated to be able to show the non-inferiority of single-dose AP compared to 3-day AP with at least 80% power (1 – *β* = 0.8) at a significance level of *α* = 5%, applying a 1:1 randomisation scheme. The non-inferiority margin was determined in order to preserve 70% of the effect of usual care on the primary outcome. For an assumed event rate of 9% in both treatment arms, this resulted in a non-inferiority margin of 4.4% (i.e. 13.4% to 9%). To prove non-inferiority, a total of 1574 patients should be recruited, in order to have 1416 evaluable patients. The study is supported by the Swiss National Science Foundation.

**Discussion:**

For AP in TURP and GL, there is a large gap between usual clinical practice and evidence-based guidelines. If single-dose AP proves non-inferior to prolonged AP, our study findings may help to reduce the duration of AP in daily routine—potentially reducing the risk of emerging resistance and complications related to AP.

**Trial registration:**

Clinicaltrials.gov, NCT03633643. Registered 16 August 2018.

**Electronic supplementary material:**

The online version of this article (10.1186/s13063-019-3237-3) contains supplementary material, which is available to authorized users.

## Background

Transurethral resection of the prostate (TURP) is one of the most frequently performed urological procedures and is associated with inpatient antibiotic use [1]. Photoselective vaporisation with the Greenlight laser (GL) has become an important therapeutic alternative to TURP, in particular for patients under anticoagulation [2]. According to the World Health Organization (WHO), TURP belongs to the category of “clean-contaminated” operative procedures [3]. Therefore, routine antimicrobial prophylaxis (AP)—ideally a single dose of the trimethoprim/sulfamethoxazole combination (TMP/SMX; cotrimoxazole is the combination product) with or without amino-penicillin/beta-lactamase inhibitor or cephalosporins—is recommended by the European Association of Urology (EAU) guidelines [4].

The bases of these guidelines are several meta-analyses [5–7] assessing the effects of various AP schemes compared to placebo. A systematic review and meta-analysis published in 2013 including a total of 42 clinical trials (randomised and quasi-randomised; 7496 patients) indicated that, in urological surgery, AP versus placebo substantially reduced the risk for bacteriuria (risk ratio (RR) 0.36, 95% confidence interval (CI) 0.29 to 0.46), urinary tract infections (UTIs) (RR 0.38, 95% CI 0.28 to 0.51), bacteraemia (RR 0.43, 95% CI 0.23 to 0.82) and fever above 38.5 °C (RR 0.41, 95% CI 0.23 to 0.73). This and the other afore-mentioned meta-analyses indicate that AP is superior compared to no AP for patient-relevant clinical outcomes (e.g. UTI, fever, sepsis) as well as for laboratory outcomes (e.g. bacteriuria, bacteraemia) [5–7].

Henceforth, to reduce postoperative UTIs after TURP, single-dose AP is recommended by the EAU guidelines [4]. A similar non-invasive intervention for prostatic hyperplasia is GL. There are, to our knowledge, no international guideline recommendations for AP though the surgical techniques and the knowledge about antimicrobial resistance have evolved, there still is overuse of antimicrobial agents for AP in TURP and GL of the prostate. In a preliminary study, we observed a non-adherence in TURP and GL to the recommended single-dose AP of more than 70% amongst urologists in Switzerland, Germany and Austria. AP in TURP and GL was regularly extended up to several days; the most common duration of AP was 2–3 days [8].

## Clinical evidence to date

In a systematic literature search in PubMed (last search 20 March 2018; detailed search strategy listed in Additional file 1, page 58), we identified five RCTs which assessed the efficacy of single-dose AP compared to prolonged AP (2–4 days or until catheter removal) of the same therapeutic compound. The largest study was conducted by Hargreave et al. [9] between 1987 and 1989 in nine European centres. Patients were randomised to receive single-dose ceftazidime (*n* = 257), continuing daily ceftazidime until catheter removal (*n* = 264) or no AP at all (*n* = 274). The study was designed so that a difference of 9% in UTIs could be detected between the non-AP group and the two AP groups. UTIs were observed significant more frequently in patients who received no AP (33.9%; 83 of 245) compared to single-dose AP (18.7%; 45 of 240) or continuous AP (11.6%; 29 of 250). Furthermore, single-dose AP resulted in significantly more UTIs compared to continuous AP. Another study, which was published in 1984, randomised patients to receive either a single dose of cefotaxime (1 g) or several doses over a 48-h regime (cefotaxime 500 mg every 12 h) [10]. From the 106 patients who received a single dose, 47 had at least one complication; while in the 48-h regime, only 24 of 97 patients had any complications. It remained unclear how complications were assessed. In an RCT published in Japanese, Tsugawa et al. [11] randomised patients to either a single dose of cefazoline (*n* = 92) or a 3-day course of cefazoline (*n* = 96). No difference in fever incidence (1-day AP, 3.3%; 3-day AP, 4.2%) or mean days until normalisation of urine analysis (1-day AP, 68.4 days; 3-day AP, 68.6 days) was observed. Two RCTs conducted by Hall et al. [12] and Costa [13] had relatively small sample sizes. Hall et al. randomised patients to either an oral dose of fleroxacin (*n* = 28), a single intravenous (IV) dose of fleroxacin (*n* = 29) or an initial IV dose of fleroxacin followed by daily oral fleroxacin until catheter removal but maximally until 5 days. No difference in the rate of UTIs (including urosepsis and fever) was observed among treatment arms (7.1% (2/28), 6.8% (2/29) and 7.4% (2/27)) in the first 6 weeks after surgery [12]. In the other RCT, men received a single perioperative dose of lomefloxacin (*n* = 20) or perioperative lomefloxacin plus daily lomefloxacin thereafter for 3 days (*n* = 20) [13]. A total of two patients and one patient developed bacteriuria in the single AP group and in the 3-day AP group, respectively. Adverse events (AEs) (e.g. nausea, vomiting and headache) seemed to be more common in the 3-day AP group.

In summary, the identified RCTs were all relatively old (published between 1984 and 1998) and none assessed the currently recommended drug combination TMP/SMX (i.e. cotrimoxazole). Furthermore, the heterogeneity among the studies was high and several studies did not assess patient-relevant outcomes.

A search on the International Clinical Trials Registry Platform (ICTRP) from the WHO (last search 20 March 2018) was conducted to assess whether there are currently ongoing RCTs which assess the impact of single-dose AP compared to prolonged AP for TURP and GL. One RCT was identified (CTRI/2017/09/009721) which plans to evaluate the efficacy of 1 day of amikacin compared to 3 days in patients undergoing TURP. For this identified trial, the target sample size is 334 patients and the primary outcome is the rate of bacteriuria 4 days after TURP.

In this multicentre RCT, we investigate the non-inferiority of single-dose AP with cotrimoxazole against 3-day AP with cotrimoxazole in terms of a patient-relevant outcome (i.e. the proportion of UTIs within 30 days, which require antimicrobial treatment) in patients undergoing either TURP or GL for obstructive voiding disorders.

## Methods

### Study design

**Fig. 1:**
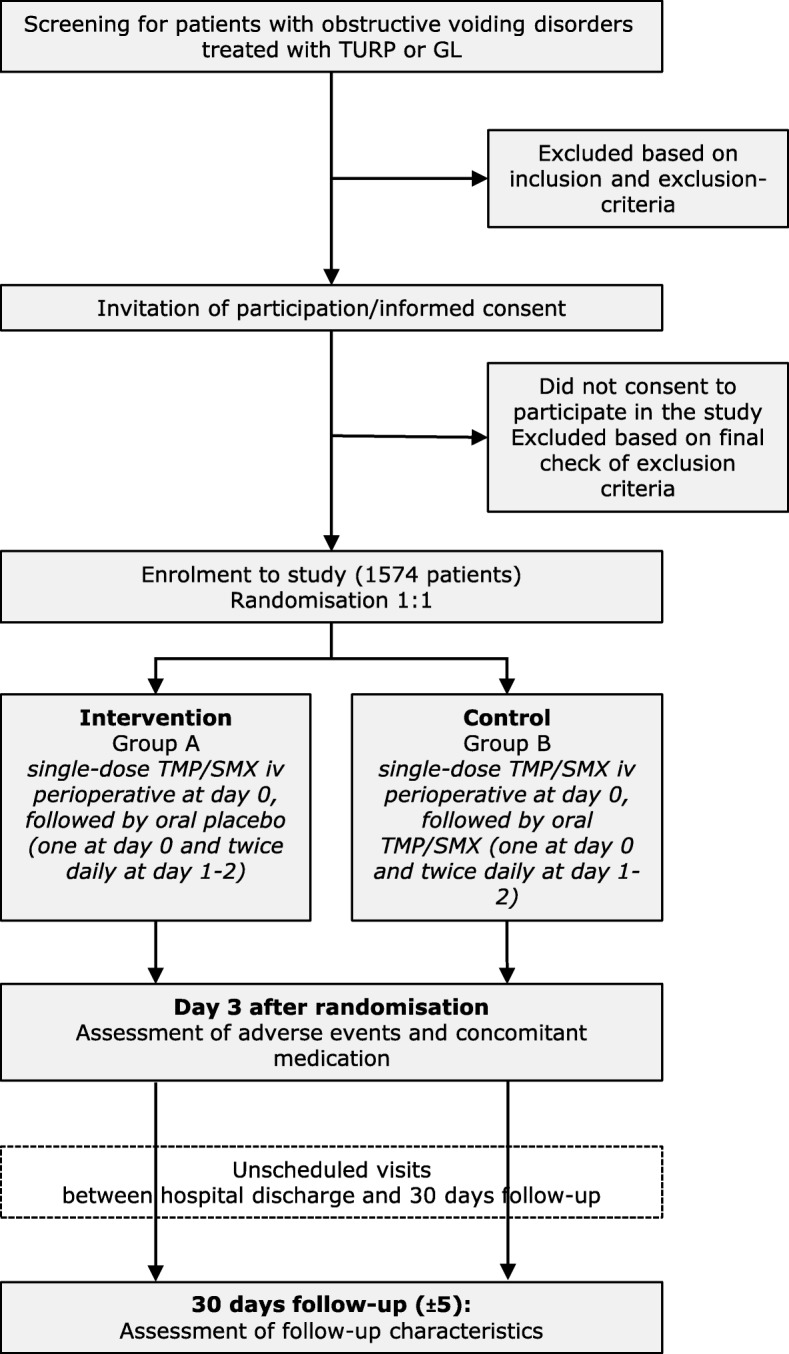
Flowchart of study design. GL Greenlight laser vaporisation, iv intravenous, TMP/SMX trimethoprim/sulfamethoxazole (cotrimoxazole), TURP transurethral resection of the prostate

This is a randomised controlled, non-inferiority, parallel group, double-blinded trial with a 1:1 randomisation ratio in five urological departments in Switzerland (i.e. University Hospital Basel, St. Claraspital Basel, University Hospital Zurich, Cantonal Hospital Aarau, Cantonal Hospital Baselland). Study participants will either be randomly allocated to guideline-conforming single-dose AP (Group A) or to 3-day AP which is currently considered usual clinical care in Switzerland (Group B). The trial will be based on routinely collected data during TURP and GL of the prostate until day 30 after surgery (Fig. 1).

### Patients

Patients scheduled for TURP or GL due to voiding disorders (e.g. benign prostate hyperplasia, obstructive prostate cancer) at the five study sites in Switzerland who meet the eligibility criteria (Table 1) will be asked to participate. Informed consent will take place at the outpatient clinic, when the surgery is planned.

**Table 1:** Inclusion and exclusion criteria

Inclusion criteria	Exclusion criteria
• Adult male patients (≥18 years) • Obstructive voiding disorder (e.g. benign prostate hyperplasia, obstructive prostate cancer) • Planned TURP or GL	• Evidence for (catheter-associated) UTI, with or without antibiotic treatment in the last 7 days prior to randomisation • Any evidence of a history of positive urine culture (cfu ≥ 10^5^/ml in midstream urine with no more than two species) and resistance to TMP/SMX in the last 7 days prior to randomisation [24] • Known contraindication against study drugs according to the Swissmedic package leaflet (e.g. patients with known liver dysfunction, renal insufficiency or glomerular filtration rate (calculated by the MDRD or CKD-EPI) < 30 ml/min or dialysis patients will be excluded) • Antibiotic treatment for any reason within 7 days prior to randomisation • Indication for AP for other reasons (e.g. endocarditis prophylaxis, transplanted patients under systemic immunosuppression)

*AP* antimicrobial prophylaxis, *cfu* colony forming units, *CKD-EPI* Chronic Kidney Disease Epidemiology Collaboration, *GL* Greenlight laser vaporisation, *MDRD* Modification of Diet in Renal Disease, *TMP/SMX* trimethoprim/sulfamethoxazole (cotrimoxazole), *TURP* Transurethral resection of the prostate, *UTI* urinary tract infection

### Outcomes

All outcomes are events within 30 days (±5 days) after randomisation if not otherwise stated.

#### Primary outcome

Symptomatic UTI (based on clinical diagnosis) treated with antibiotics (as per clinical judgement of the treating physician).

#### Secondary outcomes

- Symptomatic UTI (based on clinical diagnosis and judgement of the treating physician supported by measured bacteriuria of ≥10^5^ colony forming units (cfu)/ml) treated with antibiotics (key secondary outcome).
- Symptomatic cystitis (based on clinical diagnosis).
- Symptomatic epididymitis (based on clinical diagnosis).
- Symptomatic pyelonephritis (based on clinical diagnosis).
- Symptomatic prostatitis (based on clinical diagnosis).
- Symptomatic urethritis (based on clinical diagnosis).
- Urosepsis (based on clinical diagnosis).
- Prescription of antibiotics (for any reason).
- Asymptomatic bacteriuria of ≥10^5^ cfu/ml treated with antibiotics.
- Detection of multidrug-resistant bacteria in urine culture (three multi-resistant Gram-negatives (MRGN), 4MRGN).
- Any *Clostridium difficile*-associated infection.
- Re-hospitalisation (within 30 days after randomisation).
- All-cause mortality.
- Duration of catheterisation (cumulative sum of days between randomisation and end of catheterisation or day 30).
- Duration of hospital stay (cumulative sum of hospital days between randomisation and day 30).
- Duration of intensive care unit stay (cumulative sum of ICU days between randomisation and day 30).
- Prescribed defined daily doses (DDD) of antibiotics (cumulative sum of DDD from randomisation to day 30).
- Change of International Prostate Symptom Score (prior to surgery and at day 30 after randomisation) [14].
- Change of Quality of life Score (prior to surgery and at day 30 after randomisation) [14].

#### Safety outcomes

- Adverse events (AEs), including adverse events of special interests (i.e. diarrhoea, nausea, vomiting, allergic reaction and neurological disorder).
- Serious adverse events (SAEs), defined as any untoward medical occurrence that results in death, is life-threatening, results in re-hospitalisation or results in persistent or significant disability/incapacity.

### Randomisation

Patients will be randomly allocated to one of the treatment arms in a 1:1 ratio. Randomisation will be performed via the electronic data capture (EDC) system, which is accessible via a standard Internet browser. The investigators will enter patient details into the electronic case report form (eCRF) via a secure web interface before randomisation takes place. The randomisation procedure will include a variance minimisation algorithm which will ensure that the treatment arms are balanced for some potential confounder variables, specifically centre and surgery type (TURP or GL). In order to avoid predictable alteration of treatment allocation, and thus potential loss of allocation concealment, patients will be allocated with a probability of 0.80 to each treatment group that would minimise the difference between the groups on the key prognostic factor.

### Intervention

On the day of surgery, the anaesthetists (not related to the study) and the study nurse will be informed via the hospital information system that the patient is included in the study and that TMP/SMX (two ampoules of TMP/SMX 400/80 mg solved in 250 ml sodium chloride short infusion) has to be used as AP.

After surgery, the nurses will be informed via the hospital information system that the patient needs to receive the oral study medication. Patients randomised to Group A will receive an oral placebo on the evening of the surgery and thereafter twice daily on days 1 and 2 after randomisation (after breakfast and dinner). Patients randomised to Group B will receive oral TMP/SMX (Nopil forte®) 800/160 mg on the evening of the surgery and thereafter twice daily on day 1 and 2 after randomisation (after breakfast and dinner).

### Blinding

Physicians, patients and outcome assessors will be blinded. The manufacturing and blinding of the study medication will be performed by the Hospital Pharmacy of the University Hospital Basel according to Good Manufacturing Practice. Each study medication package consists of either five tablets of placebo (Group A) or five tablets of TMP/SMX (Nopil forte®) 800/160 mg using a licensed product repacked in a new primary packaging which is blinded.

The 500 mg placebo tablets, purchased from Fagron GmbH & Co., are optically similar but not identical to TMP/SMX (Nopil forte®) 800/160 mg. Perfectly identical placebos were not available. The blinding is ensured via non-transparent primary packaging. After randomisation, the study team will provide the corresponding medication number to a nurse who is responsible for drug distribution. This nurse will bring the study medication to the patient and instruct the patient when to take the tablets.

Patients will then take the study drug by themselves. The nurse will regularly ask whether the study drug was taken and will document this information in the hospital information system. If an unblinding due to safety concerns is required, local investigators and the delegated study personnel can decide on unblinding. Otherwise, the EDC software secuTrial® allows for unblinding by selected users (as defined in the data management plan). Each unblinding will automatically be documented in secuTrial®. Unblinding by opening the re-sealed study medication is prohibited but the possibility to do so cannot be ruled out.

### Study visits

**Fig. 2:**
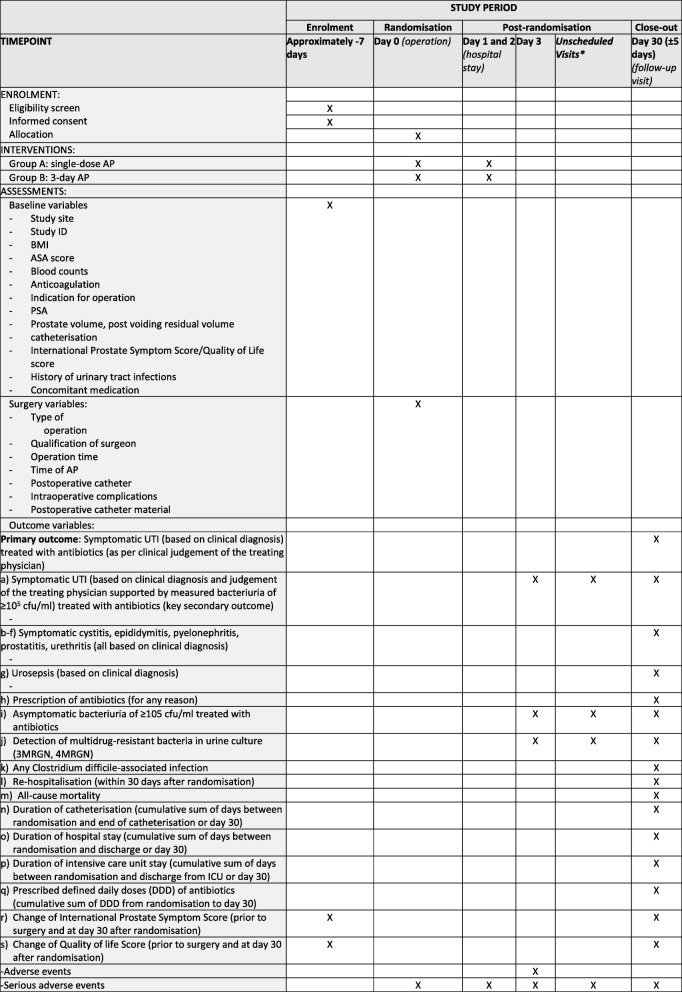
Study procedures and assessments. AP antimicrobial prophylaxis, ASA American Society of Anaesthesiologists, BMI body mass index, cfu colony forming units, DDD defined daily doses, ID identification (number), ICU intensive care unit, MRGN multi-resistant Gram-negatives, PSA prostate specific antigen, UTI urinary tract infection. *Between hospital discharge and close-out visit

An overview of all study visits and the conducted study procedures and assessments is presented in Fig. 2. During the entire duration of the study, all SAEs are collected, fully investigated and documented in source documents and eCRFs.

#### Enrolment

For each patient at enrolment (approximately 7 days before surgery), the eligibility screen, informed consent and assessment of baseline variables (study site, study ID, BMI, ASA score, blood counts, anticoagulation, PSA, prostate volume, post-voiding residual volume, catheterisation, International Prostate Symptom Score/Quality of Life score, history of UTIs) are performed by the physician who is also doing the informed consent talk for the planned surgery.

#### Allocation

Before the randomisation takes place, each patient will undergo a final eligibility check based on all available evidence from the laboratory at that time point and based on asking the patient (i.e. evidence for UTI, cfu ≥ 10^5^/ml or antibiotic treatment in the last 7 days before surgery). If all questions can be answered with no (i.e. no evidence for UTI, for cfu ≥10^5^/ml or for antibiotic treatment in the last 7 days before surgery), the patient will be randomised and will receive an identification code number via eCRF. The assessed parameters from the surgery are listed in Fig. 2. In the evening after the operation, the patients will receive a drug container with the study medication (i.e. five tablets). They will be instructed to swallow one tablet on the evening of day 0 and the consecutive four tablets on day 1 and day 2 (always one after breakfast and one after dinner).

#### Days 1 and 2

Nurses will remind the patients about the intake of each tablet.

#### Day 3

At day 3, a urine culture (with antimicrobial resistance pattern) is performed. Furthermore, AEs of special interest as well as other potential AEs will be assessed. The study nurse will acoustically check whether the container with the study medication is empty. Drug containers which are not empty will be sent back to the Hospital Pharmacy of the University Hospital Basel where the remaining tablets will be counted and destroyed by an unblinded employee, who is not further involved in the study. The eCRF contains a form to enter the number of remaining tablets, which is only accessible for users of the Hospital Pharmacy of the University Hospital Basel.

#### Unscheduled visits

Any unscheduled visits occurring during hospital discharge will be assessed at the 30-day follow-up by consulting the hospital information system and by asking the patient.

#### Follow-up after 30 days

All endpoints will be assessed at day 30 (±5 days; for more details, see “Assessment of outcomes”). In the case that a patient does not attend the 30-day visit, the treating physician or the study nurse will try to find an alternative date within the 5-day window. If this is not possible, the requested information for the primary endpoint will be asked by telephone.

### Assessment of outcomes

The vast majority of outcomes will be assessed within the routine follow-up at 30 days using routinely collected data from the hospital information system. Additionally, patients will be asked at the 30-day follow-up visit whether any symptoms occurred since the hospital discharge, whether symptoms were potentially related to a UTI (i.e. flank pain, suprapubic pain, urgency, frequency, fever), whether a physician was consulted and whether antibiotics were taken. If any of these questions are answered with yes (or if the answer remains unclear/doubtful), the information will be confirmed by contacting the treating physician.

We generally assume that the hospital information system at each study site provides highly accurate information. However, we will evaluate the agreement of data captured via the hospital information system and actively collected data (e.g. by confirmation from the treating physician) to understand the accuracy and reliability of these data. In case there is any disagreement between the data sources, the physician who conducted the 30-day follow-up (or in case of no appearance, who was supposed to conduct the 30-day follow-up) will document and resolve these discrepancies.

#### Assessment of primary outcome

The primary outcome will be assessed at the routine control 30 days after randomisation. The following procedure will be followed:

1. Before the patient arrives, the clinician who will perform the routine control or the study nurse will consult the hospital information system and assess whether the primary endpoint occurred during the hospital stay or whether the patient was re-hospitalised due to a symptomatic UTI (based on clinical diagnosis) treated with antibiotics.
2. During the routine control, the clinician will check whether the primary endpoint is currently present and, additionally, the patient will be asked whether they received any antibiotics or had a diagnosis of a UTI between hospital discharge and the routine control.
3. In the case that the patient reports that either an antibiotic was taken or such a diagnosis was made (and also if any uncertainty about the non-occurrence of the primary endpoint exists), the following clinician will be contacted:
  - the treating physician (typically the patient’s general practitioner), or
  - the corresponding physician who prescribed the antibiotics or made the diagnosis to confirm the primary endpoint.

In case the patient misses the follow-up routine control at 30 days, the routine control will be re-scheduled (the date must not be longer than 35 days after randomisation). If this is not possible, information following the same procedure as earlier (points (1)–(3)) is followed but the patient is called via telephone and asked about the occurrence of the primary endpoint.

#### Assessment of secondary outcomes

The detailed assessment of the secondary outcomes is described in the full study protocol, which was approved by the research ethical committee (see Additional file 1).

#### Assessment of safety outcomes

To assess any potential harms, the study team evaluates SAE according to the ICH E2A guidelines [15] as “definitely”, “probably”, “possibly”, “unlikely” and “not related”. The “Common Terminology Criteria for Adverse Events”, Version 5.0 terminology is going to be used for (S)AEs occurring throughout this study: 1 = mild (does not influence activities of daily living), 2 = moderate (influences activities of daily living), 3 = severe (makes some activities of daily living impossible), 4 = life-threatening, 5 = death.

#### Assessments in participants who prematurely stop the study

Enrolled patients always have the opportunity to withdraw from the trial at any time without mentioning any specific reason. In the case of a severe adverse reaction, the study drug will be stopped immediately and necessary treatment will be performed. In the case of any complication that requires antimicrobial therapy, the study drug will be stopped. Participations who stop the study drug or withdraw consent will be asked whether they are anyway willing to conduct the routine follow-up visit 30 days after surgery.

### Concomitant care

Concomitant interventions are allowed whenever necessary as part of the usual care which is based on the clinical judgement of the physician. Concomitant medications of special interest that patients take regularly at baseline are non-steroidal anti-inflammatory drugs (NSAIDs), anticoagulation, antiplatelet drugs and immunosuppressive drugs (e.g. steroids). Concomitant medications of special interest within the first 3 days after randomisation are NSAIDs.

### Sample size calculation

The primary objective is to evaluate whether single-dose AP with TMP/SMX is non-inferior to 3-day AP with TMP/SMX (i.e. usual clinical care) regarding event rates of symptomatic UTIs. The non-inferiority margin is defined as the absolute difference in the event rates. The sample size was estimated to be able to show the non-inferiority of single-dose AP compared to 3-day AP with at least 80% power (1 – β = 0.8) at a significance level of α = 5%, applying a 1:1 randomisation scheme. A drop-out rate of 10% was considered.

For determination of the non-inferiority margin *δ*, there are no direct data available describing the effect of 3-day AP with TMP/SMX versus placebo on our primary endpoint. Based on internal data from the involved clinics (see Additional file 1, page 48), we assumed an expected UTI rate within 30 days under usual care (3-day AP) of 0.09 (9%). A comprehensive meta-analysis [5] reported an overall risk ratio (RR) for symptomatic UTIs treated with AP versus placebo of 0.38. Applied to an expected UTI rate under 3-day AP of 9%, this corresponds to an expected (hypothetical) UTI rate under placebo of 23.68% (i.e. 0.2368 × 0.38 = 0.09) and an expected absolute treatment effect (absolute risk reduction (ARR)) of 3-day AP versus placebo (M1) of 14.68% (ARR = 0.2368 – 0.09 = 0.1468). The non-inferiority margin was determined in order to preserve 70% of M1 (i.e. ARR × 0.7; 10.28% on an absolute scale), which was judged a clinically important fraction. This would correspond to an absolute event rate under 1-day AP versus placebo of 13.4% (i.e. 0.2368 –0.1028 = 0.134). Assuming an event rate of 0.09 (9%) in both treatment arms, this resulted in a non-inferiority margin of 4.4% (i.e. 13.4% to 9%). For an assumed event rate of 9% in both treatment arms, and a non-inferiority margin of 4.4%, a total of 1574 patients should be recruited, in order to have 1416 evaluable patients (for more details, see Additional file 1, pages 48–49).

### Statistical analyses

The difference in the proportion of UTIs (primary outcome) between the single-dose AP and the 3-day AP arm will be compared with the non-inferiority margin using a two-sided 95% confidence interval calculated according to the continuity-corrected modification of Wilson’s score method [16]. The primary analysis will be based on the intention-to-treat (ITT) principle (i.e. all patients will be analysed according to the treatment group they were allocated to). We will also conduct a per-protocol (PP) analysis including patients without any major protocol deviation and for which the endpoint data are available. Patients who miss more than one oral dose of study medication or who did not receive the intravenous TMP/SMX due to any reasons will be classified as non-adherent to the study drug and will be excluded from the per-protocol analysis. We expect a very low rate of non-adherence to the treatment regimens in both groups due to the nature of the intervention. Thus, we do not expect substantial differences between the ITT and PP analyses. We will base our interpretation on the ITT analysis under close consideration of the results from the PP analysis when reporting and communicating the results (for more details, see Additional file 1, page 51). For the ITT analysis, missing values will be imputed via multiple imputation using fully conditional specifications (FCS) implemented by the MICE algorithm as described by van Buuren and Groothuis-Oudshoorn [17]. As a sensitivity analysis, the ITT analysis will be repeated using the inverse probability of censoring weighting (IPCW) methodology [16]. The difference in the UTI rate between the treatment arms will be estimated and reported with a 95% confidence interval separately for patients treated with TURP and patients treated with GL. No other subgroups are pre-specified. No interim analyses will be conducted.

Secondary analyses will be exploratory in nature and aim to measure the ITT effect. All estimates will be presented with 95% confidence intervals. Secondary analyses will be performed primarily as complete case analyses. For the key secondary endpoint (a) and secondary endpoints (r) and (s), additional (sensitivity) analyses will be performed, as described for the primary endpoint. For all other secondary endpoints, missing data will only be imputed or IPCW applied if the amount of missing values is considerable (i.e. more than 5% of values missing in one treatment group, more missing values than events in one group). This means that the number of patients may vary among analyses.

The key secondary outcome (a) (symptomatic UTI (based on clinical diagnosis supported by measured bacteriuria of ≥10^5^ cfu/ml) treated with antibiotics) will be analysed as described for the primary endpoint. Results (size and direction of the effect) will be compared informally to the primary endpoint.

Secondary outcomes (b)–(m) will be analysed with generalised linear mixed models (GLMM) with binomial error distribution, including the treatment arm as predictor and the study centre as random effect. Estimates will be reported as odds ratios.

Secondary outcomes (n)–(p) will be treated as count data and analysed with GLMM with Poisson error distribution, including the treatment arm as predictor and the study centre as random effect. Estimates will be reported as relative effects.

Secondary outcomes (r)–(s) will be analysed by means of linear mixed models (LMM), including the treatment arm as predictor and the study centre as random effect. Estimates will be reported as absolute effects.

### Data management and confidentiality

The data for the endpoints will be extracted from the hospital information system and entered into the eCRF by trained study nurses. Data entered into the eCRF will be validated for completeness and discrepancies automatically by implemented rules in the eCRF. An audit trail will maintain a record of initial entries and any changes made. For each patient enrolled, an eCRF must be completed. The principal investigator and co-investigator at the study site will be responsible for assuring that the data entered into the eCRF are complete and accurate, and that the entry and updates are performed in timely manner. A delegated person from the sponsor/principle investigator (i.e. Kathrin Bausch) and a designated study monitor from the Clinical Trial Unit (CTU) Basel will conduct a site initiation visit at each study site to verify the qualifications of the local investigators, inspect the site facilities and inform the investigators of their responsibilities and the procedures for ensuring adequate and correct documentation and use of the EDC system. In addition, the study monitor from the CTU Basel will conduct two routine monitoring visits per site—the first after inclusion of one or two participants, the second after inclusion of the last participant—as well as a site closure visit at the end of the study to resolve any remaining queries.

Source data must be available at the study site to document the existence of the study participants. Source data must include the original documents related to the study, as well as the medical treatment and medical history of the participant. The study eCRF will be locked after all data were entered or transferred. The complete dataset will be exported and sent to the sponsor/principle investigator via a secured protocol and according to standard operation procedures (SOPs) of the CTU Basel. All study data will be archived for a minimum of 10 years after study termination or premature termination of the trial. The study documentation and the source data will be accessible to auditors/inspectors (e.g. audits from competent authorities) and questions will be answered during inspections. All involved parties must keep the patient data strictly confidential.

## Discussion

Antimicrobial resistance is particularly prevalent among the main pathogens of the urogenital tract [18, 19]. Isolates from urological patients show high antimicrobial resistance rates. This has been explained by the frequent and extended use of antimicrobial agents partly for AP in standard urological procedures [20]. Resistance rates have been increasing in parallel with the use of antimicrobials [21]. Thus, guidelines should ensure that AP is reduced to a minimum without increasing the postoperative complications for individual patients. Recently, EAU guidelines were adapted in recommending no more explicit agents but suggesting consideration of local pathogen prevalence and preoperative urine culture in order to reduce the use of antimicrobials [22]. In 2017, 75.6% and 79.0% of *Escherichia coli* were susceptible to TMP/SMX and fluoroquinolones in north-western Switzerland [23]. Fluoroquinolones have a high propensity for collateral damage (i.e. ecological, AEs) and therefore should be reserved for severe infections other than AP or uncomplicated UTIs. In UTI treatment, their use should be limited to patients who are allergic or resistant to TMP/SMX [20].

Only a randomised controlled trial would be able to provide reliable evidence to guide decision-making on whether single-dose AP is non-inferior compared to 3-day AP in this setting. Non-randomised observational methods would carry a high risk of bias and would not allow one to draw causal inferences about the comparative merits of both treatment strategies. Therefore, in this multicentre RCT, we will investigate, in patients undergoing either TURP or GL for obstructive voiding disorders, the non-inferiority of single-dose AP with cotrimoxazole against 3-day AP with cotrimoxazole in terms of the proportion of UTIs within 30 days which require antimicrobial treatment. The goal would ultimately be to reduce AP without increasing the rate of symptomatic UTIs treated with antibiotics. We chose to extract the vast majority of endpoints (e.g. UTI, urosepsis, prostatitis) from routinely collected data without interfering much with the clinical routine to follow a pragmatic approach which would have a high external validity. A non-inferiority margin was chosen to preserve at least 70% of the original effect. In other words, when 100 patients undergoing TURP or GL would receive no treatment, 24 (i.e. 23.68%) would have a symptomatic UTI and 76 patients not. If all of these 100 were treated with 3-day AP, 9 (i.e. 9%) would still have a symptomatic UTI and 91 not. If all of these 100 were treated with a single AP (which still has 70% of the effect), approximately 13 patients would have a symptomatic UTI and 87 not. We would consider it acceptable when approximately four more of 100 patients (13 patients instead of 9) would have a UTI when bearing in mind that these four UTIs can be treated relatively straightforward with antibiotics and that simultaneously the use of AP can be strongly decreased in all 100 treated patients. This may reduce individual adverse events and development of resistant pathogens. Furthermore, lesser antibiotic resistance has important beneficial consequences for public health in general.

## Trial status

The recruitment of patients (protocol version 1.1 11.9.2018, supplement 1) has started after the submission of the study protocol to *Trials*. At the time point of conducting revisions (14–22 January 2019) we have so far enrolled 26 patients. We plan to enrol all patients from 1 November 2018 until 31 March 2022.

## Additional files

Additional file 1: Clinical study protocol. (DOCX 638 kb)

Additional file 2: World Health Organization Trial Registration Data Set. (DOCX 23 kb)
