# Supplementary material for: Single-dose versus 3-day cotrimoxazole prophylaxis in transurethral resection or greenlight laser vaporisation of the prostate: study protocol for a multicentre randomised placebo controlled non-inferiority trial (CITrUS trial)
Source: Trials. 2019 Feb 19;20:142. doi: 10.1186/s13063-019-3237-3 (PMC6381623; doi:10.1186/s13063-019-3237-3)
Supplement: Supplementary file 1 — Clinical study protocol. (DOCX 638 kb) [file 13063_2019_3237_MOESM1_ESM.docx]

Clinical Study Protocol

Single-Dose Versus 3-Day Cotrimoxazole Prophylaxis in Transurethral Resection or Greenlight Laser Vaporisation of the Prostate: A Pragmatic, Multicentre Randomised Placebo Controlled Non-Inferiority Trial

Short Title: Cotrimoxazole Prophylaxis in Transurethral Resection or Greenlight Laser Vaporisation of the Prostate

| Study Type: | Clinical trial with Investigational Medicinal Product (IMP) |
| --- | --- |
| Study Categorisation: | Clinical Trial, Category B |
| Study Registration: | Planned: clinicaltrials.gov |
| Study Identifier: | CITrUS (single-dose versus 3-day CotrImoxazole prophylaxis in TransUrethral resection of the prostate, Switzerland) |
| Sponsor:  Principal Investigator/Sponsor Investigator: | University Hospital Basel  Prof. Dr. med. Widmer, Andreas F.  Division of Infectious Diseases and Hospital Epidemiology  University Hospital Basel  Petersgraben 4  4031 Basel  Switzerland  Phone: +41 61 265 38 51  E-Mail: [andreas.widmer@usb.ch](mailto:andreas.widmer@usb.ch) |
| Investigator  Investigational Product: | PD Dr. med. Seifert, Hans-Helge  Department of Urology, University Hospital Basel, Switzerland  Dr. med. Bausch, Kathrin  Department of Urology, University Hospital Basel, Switzerland  Dr. med. Roth, Jan A.  Division of Infectious Diseases and Hospital Epidemiology, University Hospital Basel, Switzerland  Trimethoprim/Sulfamethoxazole; combination product Cotrimoxazole (Nopil forte^®^ (oral), Bactrim Inf Konz^®^ (intravenous)) |
| Protocol Version and Date: | Version 1.1, 11.09.2018 |

CONFIDENTIAL

The information contained in this document is explicitly not confidential.

Signature Pages

| Study Number | After receiving approval from the competent authorities, this study will be registered on clinicaltrials.gov, and Koordinationsstelle Forschung am Mensch (kofam). |
| --- | --- |
| Study Title | Single-Dose Versus 3-Day Cotrimoxazole Prophylaxis in Transurethral Resection or Greenlight Laser Vaporisation of the Prostate: A Pragmatic, Multicentre Randomised Placebo Controlled Non-Inferiority Trial |
|  |  |

The Sponsor-Investigator and trial statistician have approved the protocol version 1.1, dated 11.09.2018, and confirm hereby to conduct the study according to the protocol, current version of the World Medical Association Declaration of Helsinki, ICH-GCP guidelines or ISO 14155 norm if applicable and the local legally applicable requirements.

Principal/Sponsor Investigator:

Prof. Dr. Andreas Widmer

Basel, 11.09.2018

| Place/Date |  | Signature |
| --- | --- | --- |

Trial Statistician:

Dr. Vogt, Deborah

Basel, 11.09.2018

| Place/Date |  | Signature |
| --- | --- | --- |

Local Principal Investigators at study sites:

I have read and understood this trial protocol version 1.1, dated 11.09.2018 and agree to conduct the trial as set out in this study protocol, the current version of the World Medical Association Declaration of Helsinki, ICH-GCP guidelines or ISO 14155 norm and the local legally applicable requirements.

| Site University Hospital Basel  Department of Urology |  |
| --- | --- |
| Local Principal Investigator  PD Dr. Hans-Helge Seifert |  |

Basel, 11.09.2018

| Place/Date |  | Signature |
| --- | --- | --- |

Local Principal Investigators at study sites:

I have read and understood this trial protocol version 1.1, dated 11.09.2018 and agree to conduct the trial as set out in this study protocol, the current version of the World Medical Association Declaration of Helsinki, ICH-GCP guidelines or ISO 14155 norm and the local legally applicable requirements.

| Site St. Claraspital, Basel Department of Urology  Local Principal Investigator  Dr. Ardelt, Peter |  |
| --- | --- |

Basel, 11.09.2018

| Place/Date |  | Signature |
| --- | --- | --- |

Local Principal Investigators at study sites:

I have read and understood this trial protocol version 1.1, dated 11.09.2018 and agree to conduct the trial as set out in this study protocol, the current version of the World Medical Association Declaration of Helsinki, ICH-GCP guidelines or ISO 14155 norm and the local legally applicable requirements.

| Site University Hospital Zurich Department of Urology  Local Principal Investigator  PD Dr. Thomas Hermanns  Zurich, 11.09.2018 |  |  |
| --- | --- | --- |
| Place/Date |  | Signature |

Local Principal Investigators at study sites:

I have read and understood this trial protocol version 1.1, dated 11.09.2018 and agree to conduct the trial as set out in this study protocol, the current version of the World Medical Association Declaration of Helsinki, ICH-GCP guidelines or ISO 14155 norm and the local legally applicable requirements.

| Site Kantonsspital Aarau  Department of Urology  Local Principal Investigator  Dr. Maciej Kwiatkowski  Aarau, 11.09.2018 |  |  |
| --- | --- | --- |
| Place/Date |  | Signature |

Local Principal Investigators at study sites:

I have read and understood this trial protocol version 1.1, dated 11.09.2018 and agree to conduct the trial as set out in this study protocol, the current version of the World Medical Association Declaration of Helsinki, ICH-GCP guidelines or ISO 14155 norm and the local legally applicable requirements.

| Site Kantonsspital Baselland  Department of Urology  Local Principal Investigator  Dr. Svetozar Subotic |  |  |
| --- | --- | --- |

Liestal, 11.09.2018

| Place/Date |  | Signature |
| --- | --- | --- |

Table of Contents

Study synopsis 11

study summary in local language 18

Abbreviations 19

Study schedule 20

1. STUDY ADMINISTRATIVE STRUCTURE 21

1.1 Sponsor, Sponsor (Principle) Investigator 21

1.2 Investigator(s) 21

1.3 Statistician ("Biostatistician") 22

1.4 Laboratory 22

1.5 Clinical Epidemiologists 22

1.6 Monitoring Institution 22

1.7 Data Safety Monitoring Committee 22

1.8 Any other relevant Committee, Person, Organisation, Institution 22

2. ETHICAL AND REGULATORY ASPECTS 24

2.1 Study registration 24

2.2 Categorisation of study 24

2.3 Competent Ethics Committee (CEC) 24

2.4 Competent Authorities (CA) 24

2.5 Ethical Conduct of the Study 24

2.6 Declaration of interest 25

2.7 Patient Information and Informed Consent 25

2.8 Participant privacy and confidentiality 25

2.9 Early termination of the study 25

2.10 Protocol amendments 25

3. Background and Rationale 27

3.1 Background and Rationale 27

3.2 Investigational Product and Indication 28

3.3 Preclinical Evidence 28

3.4 Clinical Evidence to Date 29

3.5 Dose Rationale / Medical Device: Rationale for the intended purpose in study (pre-market MD) 30

3.6 Explanation for choice of comparator (or placebo) 30

3.7 Risks / Benefits 30

3.8 Justification of choice of study population 31

4. STUDY OBJECTIVES 32

4.1 Overall Objective 32

4.2 Primary Objective 32

4.3 Secondary Objectives 32

4.4 Safety Objectives 32

5. STUDY OUTCOMES 33

5.1 Primary Outcome 33

5.2 Secondary Outcomes 33

5.3 Other Outcomes of Interest 33

5.4 Safety Outcomes 33

6. STUDY DESIGN 34

6.1 General study design and justification of design 34

6.2 Methods of minimising bias 34

6.2.1 Randomisation 34

6.2.2 Blinding procedures 34

6.2.3 Other methods of minimising bias 35

6.3 Unblinding Procedures (Code break) 35

7. STUDY POPULATION 36

7.1 Eligibility criteria 36

7.2 Recruitment and screening 36

7.3 Assignment to study groups 36

7.4 Criteria for withdrawal / discontinuation of participants 36

8. STUDY INTERVENTION 38

8.1 Identity of Investigational Products 38

8.1.1 Experimental Intervention (treatment / medical device) 38

8.1.2 Control Intervention (standard/routine/comparator treatment / medical device) 38

8.1.3 Packaging, Labelling and Supply (re-supply) 38

8.1.4 Storage Conditions 38

8.2 Administration of experimental and control interventions 38

8.2.1 Experimental Intervention 38

8.2.2 Control Intervention 38

8.3 Dose / Device modifications 38

8.4 Compliance with study intervention 39

8.5 Data Collection and Follow-up for withdrawn participants 39

8.6 Trial specific preventive measures 39

8.7 Concomitant Interventions (treatments) 39

8.8 Study Drug / Medical Device Accountability 39

8.9 Return or Destruction of Study Drug / Medical Device 39

9. STUDY ASSESSMENTS 40

9.1 Table of study procedures and assessments 40

9.2 Assessments of outcomes 41

9.2.1 Assessment of primary outcome 41

9.2.2 Assessment of secondary outcomes 42

9.2.3 Assessment of other outcomes of interest 44

9.2.4 Assessment of safety outcomes 44

9.2.5 Assessments in participants who prematurely stop the study 45

9.3 Procedures at each visit 45

See also Section 9.1. 45

9.3.1 Enrolment 45

9.3.2 Allocation 45

9.3.3 Day 1 and 2 45

9.3.4 Day 3 45

9.3.5 Unscheduled Visits 45

9.3.6 Follow-up after 30 days 45

10. SAFETY 46

10.1 Drug studies 46

10.1.1 Definition and assessment of (serious) adverse events and other safety related events 46

10.1.2 Reporting of serious adverse events (SAE) and other safety related events 47

10.1.3 Follow-up of (Serious) Adverse Events 48

10.2 Medical Device Category C studies 48

10.3 Medical Device Category A studies 48

11. STATISTICAL METHODS 49

11.1 Hypothesis 49

11.2 Determination of Sample Size 49

11.3 Statistical criteria of termination of trial 50

11.4 Planned Analyses 50

11.4.1 Datasets to be analysed, analysis populations 50

11.4.2 Primary Analysis 51

11.4.3 Secondary Analyses 51

11.4.4 Interim analyses 52

11.4.5 Safety analysis 52

11.4.6 Deviation(s) from the original statistical plan 52

11.5 Handling of missing data and drop-outs 52

12. QUALITY ASSURANCE AND CONTROL 53

12.1 Data handling and record keeping / archiving 53

12.1.1 Case Report Forms 53

12.1.2 Specification of source documents 53

12.1.3 Record keeping / archiving 53

12.2 Data management 53

12.2.1 Data Management System 53

12.2.2 Data security, access and back-up 53

12.2.3 Analysis and archiving 53

12.2.4 Electronic and central data validation 53

12.3 Monitoring 54

12.4 Audits and Inspections 54

12.5 Confidentiality, Data Protection 54

12.6 Storage of biological material and related health data 54

13. PUBLICATION AND DISSEMINATION POLICY 55

14. FUNDING AND SUPPORT 55

14.1 Funding 55

14.2 Other Support 55

15. INSURANCE 55

16. REFERENCES 56

17. APPENDICES 59

17.1 Search strategy 59

Study synopsis

| Principle / Sponsor / Sponsor Investigator | Prof. Dr. med. Widmer, Andreas F.  Division of Infectious Diseases and Hospital Epidemiology  University Hospital Basel  Petersgraben 4  4031 Basel  Switzerland  Phone: +41 61 265 38 51 |
| --- | --- |
| Study Title: | Single-Dose Versus 3-Day Cotrimoxazole Prophylaxis in Transurethral Resection or Greenlight Laser Vaporisation of the Prostate: A Pragmatic, Multicentre Randomised Placebo Controlled Non-Inferiority Trial |
| Short Title / Study ID: | Cotrimoxazole Prophylaxis in Transurethral Resection or Greenlight Laser Vaporisation of the Prostate  Acronym: CITrUS (single-dose versus 3-day CotrImoxazole prophylaxis in TransUrethral resection of the prostate, Switzerland) |
| Protocol Version and Date: | Version 1.1, 09.11.2018 |
| Trial registration: | Planned: clinicaltrials.gov, and kofam (Koordinationsstelle Forschung am Mensch) |
| Study category and Rationale | Category B (pharmaceutical product involved, no medical device or transplant involved) |
| Clinical Phase: | Phase 4; therapeutic use |
| Background and Rationale: | Increasing antimicrobial resistance rates have a substantial impact on morbidity, mortality and healthcare costs. For instance, ciprofloxacin – a frequently used antimicrobial agent in urology – has partly lost its effectiveness, which may be explained by a widespread overuse of quinolones and other antimicrobial agents in urology.  Antimicrobial prophylaxis (AP) in urological interventions such as transurethral resection of the prostate (TURP) is recommended to reduce postoperative infections – ideally as a single-dose: Several meta-analyses of randomised controlled trials (RCTs) underline the benefits of an AP compared to placebo to reduce postoperative urinary tract infections (UTI), fever, sepsis, and bacteriuria. For Greenlight Laser Vaporisation (GL) of the prostate, which has become an important alternative to TURP for patients under oral anticoagulation, no RCTs have been published comparing different AP durations or regimens. Even though the surgical techniques and the knowledge about antimicrobial resistance has evolved, there still is an overuse of antimicrobial agents for AP in TURP and GL of the prostate: In a preliminary study, we showed a non-adherence in TURP and GL (by applying TURP guidelines to GL) to the recommended single-dose AP of more than 70% amongst urologists in Germany, Austria and Switzerland: AP in TURP and GL was regularly extended up to several days; the most common duration of AP was 2-3 days, leading to change of the microflora of the patient. Therefore, for patients receiving a TURP or GL of the prostate, we plan to compare a guideline-conform single-dose AP with trimethoprim/sulfamethoxazole (TMP/SMX; i.e. Cotrimoxazole) versus usual clinical care (i.e. 3-day AP with Cotrimoxazole) in a multicentre, randomised, controlled trial to assess the non-inferiority of a single-dose AP in terms of preventing UTIs (based on clinical diagnosis), which require treatment with an antimicrobial agent.  Our study results help clinicians on the optimal duration of AP in TURP and GL. If single-dose proves to be non-inferior to prolonged “prophylaxis”, such results may shorten duration of AP, reducing the risk of emerging resistance and lower the risk for antibiotic-therapy related complications. |
| Objective(s): | Primary objective  To determine in patients undergoing a TURP or GL of the prostate if a single-dose AP with Cotrimoxazole is non-inferior to a 3-day AP with Cotrimoxazole in preventing the occurrence of symptomatic UTI (based on clinical diagnosis), which are treated with antimicrobial agents within 30 days after randomisation.  Secondary objectives  To evaluate the impact of a single-dose AP with Cotrimoxazole compared to a 3-day AP with Cotrimoxazole on further clinically and patient-relevant outcomes, including alternative definitions of the primary outcome (symptomatic UTI based on clinical diagnosis supported by measured bacteriuria of ≥10^5^ cfu/ml, treated with antimicrobial agents), symptomatic cystitis, epididymitis, pyelonephritis, prostatitis, urethritis, urosepsis (all based on clinical diagnosis) as well as prescribed antibiotics (also as prescribed defined daily doses (DDD)), asymptomatic bacteriuria (≥10^5^ cfu/ml) treated with antibiotics, detection of multidrug-resistant bacteria (3MRGN, 4MRGN), any *Clostridium difficile*-associated infection, duration of catheterisation, (re-)hospitalisation, intensive care unit (ICU) stay, change of International Prostate Symptom and Quality of Life Score (IPSS, QoL), all-cause mortality, total and (serious) adverse events (AE, SAE). |
| Outcome(s): | All outcomes are events within 30 days (±5 days) after randomisation if not stated otherwise. Primary outcome measure Symptomatic UTI (based on clinical diagnosis) treated with antimicrobial agents. Secondary outcome measuresSymptomatic UTI (based on clinical diagnosis supported by measured bacteriuria of ≥10^5^ cfu/ml) treated with antimicrobial agents (**key secondary outcome**)  - Symptomatic cystitis (based on clinical diagnosis) - Symptomatic epididymitis (based on clinical diagnosis) - Symptomatic pyelonephritis (based on clinical diagnosis) - Symptomatic prostatitis (based on clinical diagnosis) - Symptomatic urethritis (based on clinical diagnosis) - Urosepsis (based on clinical diagnosis) - Prescription of antibiotics (for any reason) - Prescribed defined daily doses (DDD) of antibiotics (cumulative sum of DDD from randomisation to day 30) - Asymptomatic bacteriuria of ≥10^5^ cfu/ml treated with antimicrobial agents - Detection of multidrug-resistant bacteria in urine culture (3MRGN, 4MRGN) - Any *Clostridium difficile*-associated infection - Duration of catheterisation (cumulative sum of days between randomisation and end of catheterisation or day 30) - Duration of hospital stay (cumulative sum of hospital days between randomisation and day 30) - Duration of intensive care unit stay (cumulative sum of ICU days between randomisation and day 30) - Re-hospitalisation (within 30 days after randomisation) - Change of International Prostate Symptom Score (prior to randomisation and at day 30 after randomisation) - Change of Quality of life Score (prior to randomisation and at day 30 after randomisation) - All-cause mortality - Total adverse events - Total serious adverse events |
| Study design: | Pragmatic, placebo controlled, parallel arm, non-inferiority, multicentre, randomised controlled trial based on routinely collected data with blinded treating physicians, patients and outcome assessors. |
| Inclusion / Exclusion criteria: | Inclusion criteria  - Adult male patients (≥18 years) - Obstructive voiding disorder (e.g. benign prostate hyperplasia, obstructive prostate cancer) - Planned TURP or GL  Exclusion criteria  - Evidence for (catheter associated-) UTI, with or without antibiotic treatment in the last 7 days prior to randomisation. - Any evidence of a history of positive urine culture (cfu ≥10^5^/ml in midstream-urine with no more than two species) and resistance to TMP/SMX in the last 7 days prior to randomisation. - Known contraindication against study drugs according to the Swissmedic package leaflet (e.g. known liver dysfunction, renal insufficiency; patients with glomerular filtration rate (calculated by the MDRD or CKD-EPI) <30ml/min or dialysis patients will be excluded). - Antibiotic treatment for any reason within 7 days prior to randomisation - Indication for AP for other reasons (e.g. endocarditis prophylaxis, transplanted patients under systemic immunosuppression). |
| Measurements and procedures: | We plan to enrol from 1^st^ October 2018 to 31^st^ March 2022. All patients at the 5 study sites meeting the previously mentioned criteria will be asked to participate and are assigned an electronic case report form (eCRF) number, which is unique and will be collected in an electronic data capture (EDC) system. Patients will be randomised at the day of the operation to either a single-dose AP with Cotrimoxazole or a 3-day AP with Cotrimoxazole. All outcomes will be assessed during clinical routine (3 days after randomisation) and during the routine follow-up 30 days (±5 days) after randomisation. Additionally, patients will be asked at the 30 day follow-up visit if any symptoms (e.g. voiding disorders, symptoms of UTI) occurred since the hospital discharge, if a physician was consulted, and if antibiotics were taken. All these information will be confirmed by contacting the treating physician and any discrepancies will be documented and resolved by the clinician conducting the 30 day follow-up. |
| Study Product / Intervention: | Group A  Single-dose TMP/SMX (i.e. Cotrimoxazole) perioperative as two ampoules of TMP/SMX 400/80 mg (Bactrim Inf Konz^®^) solved in 250 ml sodium chloride short infusion followed by five oral applications of placebo (lactose tablet; Fagron GmbH & Co. KG) at the evening of the surgery and thereafter twice daily on day 1 and 2 after surgery while the patient is in hospital. |
| Control Intervention (if applicable): | Group B  3-day AP with TMP/SMX (i.e. Cotrimoxazole): Preoperatively as two ampoules of TMP/SMX 400/80mg (Bactrim Inf Konz®) solved in 250 ml sodium chloride short infusion, followed by five oral applications of TMP/SMX 800/160 mg (Nopil forte^®^ tablets) at the evening of the surgery and thereafter twice daily on day 1 and 2 after surgery while the patient is in hospital. |
| Number of Participants with Rationale: | Sample size was estimated to be able to show the non-inferiority of single-dose AP compared to 3-day AP with at least 80% power (1 – *β* = 0.8) at a significance level of *α* = 5%, applying a 1:1 randomisation scheme. A drop-out rate of 10% was considered. The non-inferiority margin $\delta$ was determined in order to preserve 70% of the effect of usual care on the primary outcome. For an assumed event rate of 9% in both trial arms, and $\delta$ of 4.4%, a total of 1,574 patients should be recruited, in order to have 1,416 evaluable patients. |
| Study Duration: | Approximately 42 months |
| Study Schedule: | First patient in: Approximately October 2018  Last patient out: Approximately March 2022 |
| Investigator(s): | Prof. Dr. med. Widmer, Andreas F.  Division of Infectious Diseases and Hospital Epidemiology  University Hospital Basel  Petersgraben 4  4031 Basel  Switzerland  Phone: +41 61 265 38 51  E-Mail : [andreas.widmer@usb.ch](mailto:andreas.widmer@usb.ch)  PD Dr. med. Seifert, Hans-Helge  Department of Urology, University Hospital Basel, Switzerland  Dr. med. Bausch, Kathrin  Department of Urology, University Hospital Basel, Switzerland  Dr. med. Roth, Jan A.  Division of Infectious Diseases and Hospital Epidemiology, University Hospital Basel, Switzerland |
| Study Centre(s): | Departments of Urology   - University Hospital Basel, Basel, Switzerland - St. Claraspital, Basel, Switzerland - University Hospital Zurich, Zurich, Switzerland - Kantonsspital Aarau, Aarau, Switzerland - Kantonsspital Baselland, Liestal, Switzerland |
| Statistical Considerations: | Primary analysis  The difference in the proportion of UTIs between the single-dose AP and the 3-day AP arm will be compared with the non-inferiority margin using a two-sided 95 % confidence interval calculated according to the continuity-corrected modification of Wilson’s score method. The primary analysis will be based on the intention to treat (ITT) principle. We will also conduct a per protocol (PP) analysis. We expect a very low rate of non-adherence to the treatment regimens in both groups due to the nature of the intervention. Thus we do not expect substantial differences between ITT and PP analyses. We will base our interpretation on the ITT analysis under close consideration of the results of the PP analysis when reporting and communicating the results.  **Sensitivity analysis:** In order to evaluate the robustness of imputation method the ITT analysis of the primary analysis will be repeated using inverse probability censored weighting (IPCW) methodology.  **Subgroup analysis:** The difference in the UTI proportion between the study arms will be explorative assessed with 95% confidence interval for patients treated with TURP and patients treated with GL.  Secondary analyses  Analyses of all secondary outcomes will be exploratory in nature. All estimates will be presented with 95% confidence intervals. |
| GCP Statement: | This study will be conducted in compliance with the study protocol, the current version of the Declaration of Helsinki, the ICH-GCP or ISO EN 14155 (as far as applicable) as well as all national legal and regulatory requirements. |

study summary in local language

**Einzeldosis gegenüber einer Drei-Tage-Prophylaxe während einer transurethralen Resektion der Prostata oder Greenlight Laser Vaporisation der Prostata: eine pragmatische, multizentrische, placebokontrollierte Nichtunterleigenheitsstudie.**

**Hintergrund:** In urologischen Kliniken werden deutlich höhere Raten an antibiotikaresistenten Keimen als in anderen Disziplinen vorgefunden, bedingt durch den enormen Einsatz von Antibiotika. Etwa 10‘000 Männer erhalten in der Schweiz jährlich eine transurethralen Resektion der Prostata; die Greenlight Laservaporisation der Prostata hat sich als Alternative für Patienten unter blutverdünnender Therapie etabliert. Internationale Leitlinien empfehlen eine Einzeldosis einer antimikrobiellen Prophylaxe zur Vermeidung von Infektkomplikationen. Die klinische Praxis weicht jedoch stark davon ab, wodurch die Entwicklung von antimikrobiellen Resistenzen gefördert wird.

**Ziele:** In einer multizentrischen, randomisierten Studie soll untersucht werden, ob eine leitlinienkonforme einmalige antimikrobielle Prophylaxe während der transurethralen Resektion der Prostata oder der Greenlight Laservaporisation der Prostata einer dreitägigen Gabe hinsichtlich den Raten postoperativer Infektionen, die eine antibiotische Behandlung benötigen, nicht unterlegen ist.

**Methoden:** Patienten, die eine transurethrale Resektion oder Laservaporisation der Prostata erhalten, werden zufällig einer leitlinienkonformen Einzeldosisgruppe oder einer Drei-Tage-Prophylaxe Gruppe zugeteilt. An fünf Studienzentren werden u.a. postoperative Harnwegsinfekte, antibiotikaassoziierten Nebenwirkungen und die Entwicklung von antimikrobiellen Resistenzen in den ersten 30 Tagen nach der Operation untersucht. Wir gehen von einer postoperativen Infekt Rate von 9% aus und untersuchen die Nicht-Unterlegenheit der Einzeldosisgruppe hinsichtlich der Entwicklung von Harnwegsinfekten, die mit Antibiotika behandelt werden müssen.

**Bedeutung/möglicher Nutzen:** Eine einzelne Dosis für die Antibiotikaprophylaxe statt mehrere Tage führt zu einer geringeren Belastung für den Patienten, weniger Nebenwirkungen und einem geringeren Risiko der Antibiotika-Resistenzentwicklung, vorausgesetzt, dass die Infektionshäufigkeit vergleichbar bleibt. Diese vergleichbar einfache Verbesserung könnte einer hohen Anzahl von Patienten zugutekommen und könnte es erlauben bewährte Antibiotikasubstanzen auch in Zukunft in der Prophylaxe und Therapie verwenden zu können.

Abbreviations

| AE  AP  ASA  ATC  BASEC  CA  cfu  CEC  CI  CKD-EPI  CRP  CTU  DDD  EAU  EC  (e)CRF  EDC  ESCMID  GCP  GL  GLMM  ICH  ICTRP  ICU  ID  IPCW  IPSS  ITT  IV  KOFAM  LMM  MDRD  MRGN  NFP  NSAID  PP  PSA  QoL  RCT  RR  SAE  SmPC  SNF  SOP  SUSAR  TMP/SMX  TURP  UTI  WHO  WWW  °C | Adverse events  Antibiotic prophylaxis  American Society of Anesthesiologists  Anatomical Therapeutic Chemical Classification System  Business Administration System for Ethics Committees  Competent Authorities  Colony forming unit  Competent Ethics Committee  Confidence interval  Chronic Kidney Disease Epidemiology Collaboration  C-reactive protein  Clinical Trial Unit  Defined daily doses  European Association of Urology  Ethical Committees  (electronic) case report form  Electronic data capture  European Society for clinical microbiology and infection  Good Clinical Practice  Greenlight Laser  generalised linear mixed models  International Council for Harmonisation of Technical Requirements for Pharmaceuticals for Human Use  International Clinical Trials Registry Platform  Intensive Care Unit  Identification (Number)  Inverse probability censored weighting  International Prostate Symptom Score  Intention to treat  Intravenous  Koordinationsstelle Forschung am Mensch  Linear mixed models  Modification of Diet in Renal Disease  Multiresistant gram negatives  Nationales Forschungsprogramm  Non-steroidal anti-inflammatory drugs  Per protocol  Prostate-specific antigen  Quality of Life  Randomised clinical trial  Relative risk ratio  Serious adverse events  Summary of Product Characteristics  Swiss National Foundation  Standard Operation Procedure  Suspected Unexpected Serious Adverse Reaction  Trimethoprim/sulfamethoxazole  Transurethral resection of the prostate  Urinary tract infection  World Health Organization  World Wide Web  Degrees Celsius |
| --- | --- |
|  |  |

Study schedule

**
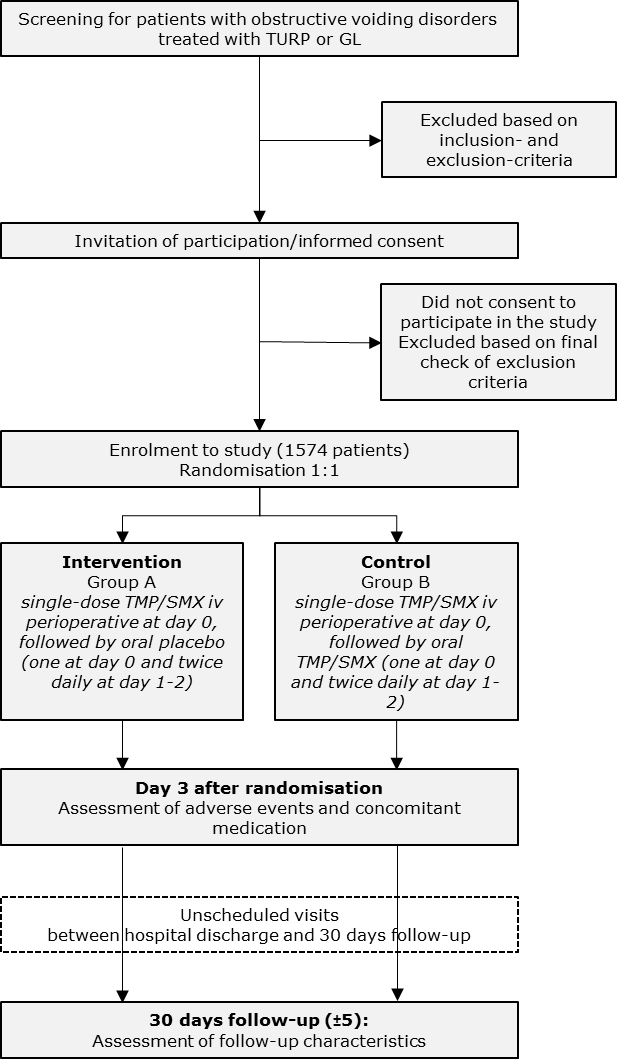
**

**Figure 1**: Flow for participants.

Abbreviations: GL, Greenlight laser vaporisation; iv, intravenous; TMP/SMX, trimethoprim/sulfamethoxazole=Cotrimoxazole; TURP, transurethral resection of the prostate.

# STUDY ADMINISTRATIVE STRUCTURE

## Sponsor, Sponsor (Principle) Investigator

Prof. Dr. med. Widmer, Andreas F.

Division of Infectious Diseases and Hospital Epidemiology

University Hospital Basel

Petersgraben 4

4031 Basel

Switzerland

Phone: +41 61 265 38 51

## Investigator(s)

PD Dr. med. Seifert, Hans-Helge

Department of Urology, University Hospital Basel, Switzerland

Dr. med. Bausch, Kathrin

Department of Urology, University Hospital Basel, Switzerland

Dr. med. Roth, Jan A.

Division of Infectious Diseases and Hospital Epidemiology, University Hospital Basel, Switzerland

Local Principle Investigator(s):

PD Dr. med. Seifert, Hans-Helge

Department of Urology

University Hospital Basel

Spitalstrasse 21

4056 Basel

Switzerland

Phone: +41 61 265 72 80

Dr. med. Ardelt, Peter

Department of Urology

St. Claraspital

Kleinriehenstrasse 30

4058 Basel

Switzerland

Phone: +41 61 685 85 23

PD Dr. med. Hermanns, Thomas

Department of Urology

University Hospital Zurich

Nord1 B 204

Frauenklinikstrasse 10

8091 Zurich

Switzerland

Phone: +41 44 255 54 40

Dr. med. Kwiatkowski, Maciej

Department of Urology

Kantonsspital Aarau

Tellstrasse 25

5001 Aarau

Switzerland

Phone: +41 62 838 47 47

Dr. med. Subotic, Svetozar

Department of Urology

Kantonsspital Baselland

Rheinstrasse 26

4410 Liestal

Switzerland

Phone: +41 61 925 21 72

## Statistician ("Biostatistician")

Dr. Vogt, Deborah

Clinical Trial Unit

Department Klinische Forschung

University Hospital Basel

Switzerland

## Laboratory

Not applicable. Routinely collected data is examined at the respective laboratories of participating study sites. All certified by Swiss Good Laboratory Practice, ISO/IEC 17025 and ISO 15189.

## Clinical Epidemiologists

Dr. Speich, Benjamin

PD Dr. med. Hemkens, Lars G.

Basel Institute for Clinical Epidemiology and Biostatistics

Department Klinische Forschung

University Hospital Basel

Switzerland

## 1.6 Monitoring Institution

Clinical Trial Unit

Departement Klinische Forschung

University Hospital Basel

Switzerland

## 1.7 Data Safety Monitoring Committee

Not applicable

## 1.8 Any other relevant Committee, Person, Organisation, Institution

Clinical Trial Unit

Departement Klinische Forschung

University Hospital Basel

Switzerland

Dr. Deuster, Stefanie (supply of study medication)

Hospital Pharmacy

University Hospital Basel

Switzerland

Swiss National Science Foundation, funder and official supporter of the study.

**1.9 Authors contributions**

Authors contributing to study protocol: Andreas F. Widmer, Hans-H. Seifert, Jan A. Roth, Benjamin Speich, Lars G. Hemkens, and Kathrin Bausch had the study idea and designed the study. Kathrin Bausch, Benjamin Speich and Lars G. Hemkens wrote the study protocol. Deborah R. Vogt planned the statistical aspects of the study and wrote the statistical analysis section with input from Lars G. Hemkens. The data management system was designed by Nicole Bruni. The monitoring system is provided by Klaus Ehrlich. Hans-H. Seifert, Thomas Hermanns, Peter Ardelt, Svetozar Subotic, Maciej Kwiatkowski read and revised the study protocol. Stefanie Deuster is responsible for providing the study medication according to Good Manufacturing Practice.

# ETHICAL AND REGULATORY ASPECTS

Before the study will be conducted, the protocol will be submitted to a properly constituted Competent Ethics Committee (CEC) and Swissmedic. The decision of the CEC and Swissmedic concerning the conduct of the study will be made in writing to the Principle/Sponsor Investigator before commencement of this study.

The study can only begin once approval from all required authorities has been received. Any additional requirements imposed by the authorities shall be implemented.

## Study registration

The trial will be registered with the Koordinationsstelle Forschung am Mensch (kofam) and the U.S. National Institutes of Health ([www.clinicaltrials.gov](http://www.clinicaltrials.gov)).

## Categorisation of study

Category B. This is a pragmatic study based on primary routinely collected health care data. The study drug is applied according to the Summary of Product Characteristics (SmPC). The intervention does not pose any additional harm to a patient because both the intervention and the control are clinical routine already. To provide adequate blinding, the study medication (i.e. Cotrimoxazole (Nopil forte^®^) and placebo) tablets will be packed and labelled according to Good Manufacturing Practice at the Hospital Pharmacy of the University Hospital Basel.

## Competent Ethics Committee (CEC)

The Principle/Sponsor Investigator will ensure that approval from an appropriately constituted Competent Ethics Committee (CEC) is sought for the study.

No changes of the protocol will be implemented, unless to prevent immediate danger, without prior Sponsor and Ethics committee approval.

Premature study end or interruption of the study will be reported within 15 days. The regular end of the study is reported to the CEC within 90 days, the final study report shall be submitted within one year after study end. Amendments are reported according to chapter 2.10.

## Competent Authorities (CA)

The protocol of this trial will be submitted to the ethical committees (EC) ‘Nordwest- und Zentralschweiz’ (Leitethikkommission) and Zürich. Furthermore, the study protocol will be submitted to Swissmedic. The completion of this study will be reported to the CAs within 90 days and the final study report will be submitted within one year. In case that the study is discontinued or interrupted, the CA will be informed within 15 days. Non-substantial amendments will be reported as soon as possible to CAs. Substantial amendments are reported according to Section 2.10.

## Ethical Conduct of the Study

The study will be carried out in accordance to the protocol and with principles enunciated in the current version of the Declaration of Helsinki, the guidelines of Good Clinical Practice (GCP) issued by ICH, in case of medical device: the European Directive on medical devices 93/42/EEC and the ISO Norm 14155 and ISO 14971, the Swiss Law and Swiss regulatory authority’s requirements. The CEC and regulatory authorities will receive annual safety and interim reports and be informed about study stop/end in agreement with local requirements.

All staff involved in the pragmatic trial will have to fulfil requirements in regard to training, data management and data analysis.

Writing of protocol and final manuscripts will be in adherence with reporting standards of SPIRIT, CONSORT and RECORD.

## Declaration of interest

This is an investigator-initiated trial conducted entirely with public support by the Swiss National Science Foundation within the Nationale Forschungsprogramm ‘Antimikrobielle Resistenz’ (NFP 72; <http://www.nfp72.ch/en/projects/module-3-optimised-use-of-antibiotics/detecting-excessive-antibiotic-use-in-urology>).

The Sponsor of the trial is the University Hospital Basel. There is no conflict of interests.

## Patient Information and Informed Consent

The investigators will explain to each participant the nature of the study, its purpose, the procedures involved, the expected duration, the potential risks and benefits and any discomfort it may entail. Each participant will be informed that the participation in the study is voluntary and that he may withdraw from the study at any time and that withdrawal of consent will not affect his subsequent medical assistance and treatment.

The participant must be informed that his medical records may be examined by authorised individuals other than their treating physician.

All participants for the study will be provided a participant information sheet and a consent form describing the study and providing sufficient information for participant to make an informed decision about their participation in the study. Enough time is given to the patients to consider participation because the information sheet and consent form are handed out at the preoperative consultation, which is scheduled approximately 1 week prior to the surgery. Therefore, it is ensured, that patients are informed more than 24 hours prior to the surgery.

The patient information sheet and the consent form will be submitted to the CEC and to the competent authority (as applicable) to be reviewed and approved. The formal consent of a participant, using the approved consent form, must be obtained before the participant is submitted to any study procedure.

The participant should read and consider the statement before signing and dating the informed consent form, and should be given a copy of the signed document. The consent form must also be signed and dated by the investigator (or his designee) and it will be retained as part of the study records.

## Participant privacy and confidentiality

The investigator affirms and upholds the principle of the participant's right to privacy and that they shall comply with applicable privacy laws. Especially, anonymity of the participants shall be guaranteed when presenting the data at scientific meetings or publishing them in scientific journals.

Individual subject medical information obtained as a result of this study is considered confidential and disclosure to third parties is prohibited. Subject confidentiality will be further ensured by utilising subject identification code numbers to correspond to treatment data in the computer files.

Ethics committees and Swissmedic have guaranteed access to all original and processed data and permission to audit the project at any time.

## Early termination of the study

The Sponsor/Principle Investigator may terminate the study prematurely according to certain circumstances, for example unexpected ethical concerns, insufficient patient recruitment, alterations in accepted clinical practice that make the continuation of a clinical trial unwise, when the safety of the participants is doubtful or at risk or early evidence of harm of the experimental intervention.

## Protocol amendments

Substantial amendments are only implemented after approval of the CAs.

Under emergency circumstances, deviations from the protocol to protect the rights, safety and well-being of human subjects may proceed without prior approval of the Sponsor/Principle Investigator and the CAs. Such deviations shall be documented and reported to the Sponsor/Principle Investigator and the CAs as soon as possible.

A formal amendment to the protocol will be made for issues that may impact the conduct of the study or affect patient’s benefit or harm. This includes particularity substantive changes of the objectives, design, eligibility criteria, sample size, and duration of follow-up. Such major amendments will be agreed upon by the study investigators and approved by the CAs prior to implementation.

Minor changes that have no effect on the study conduct of primarily administrative nature will be documented in a memorandum. The investigators will inform the CAs about such minor changes at their discretion. All changes will be documented in the final results publication of the study.

# Background and Rationale

## Background and Rationale

Transurethral resection of the prostate (TURP) is one of the most frequently performed urological procedures and is associated with inpatient antibiotic use (1). Photoselective vaporisation with the Greenlight Laser (GL) has become an important therapeutic alternative to TURP, in particular for patients under anticoagulation (2). According to the World Health Organization (WHO), TURP belongs to the category of “clean-contaminated” operative procedures (3). Therefore, routine antimicrobial prophylaxis (AP) – ideally a single-dose of the trimethoprim/sulphamethoxazole combination (TMP/SMX; Cotrimoxazole is the combination product) with or without amino-penicillin/beta-lactamase inhibitor or cephalosporins – is recommended by the European Association of Urology (EAU) guidelines (4).

Basis of these guidelines are several meta-analyses (5-7) assessing the effects of various AP schemes compared to placebo: A systematic review and meta-analysis published in 2013, including a total of 42 clinical trials (randomised and quasi-randomised; 7,496 patients) indicated that, in urological surgery, AP versus placebo substantially reduced the risk for bacteriuria (relative risk ratio (RR) 0.36, 95% CI 0.29 to 0.46), urinary tract infections (UTI) (RR 0.38, 95% CI 0.28 to 0.51), bacteraemia (RR 0.43, 95% CI 0.23 to 0.82) and fever above 38.5 degrees Celsius (°C) (RR 0.41, 95% CI 0.23 to 0.73). Until 10 days after TURP, bacteriuria occurred in 6.8% of patients using AP, sepsis in 2.9% and UTIs in 2.9% (5). Another systematic review and meta-analysis published in 2005 by Qiang et al. included a total of 28 quasi-randomised and randomised clinical trials (RCT; 4,694 patients). They investigated low risk patients without substantial preoperative bacteriuria undergoing TURP and found in patients using AP in 9.2% bacteriuria and in 2.6% fever over 38.5°C one week after surgery (6). Patients without AP had in 26.5% of all cases bacteriuria and 13.5% had fever over 38.5°C. Those findings are in line with a meta-analysis published in 2002 by Berry and Barratt, including 32 randomised clinical trials (4,260 patients): 2-5 days after TURP bacteriuria occurred in 9.1% (213/2,346) and 26.0% (497/1,914) in patients treated with and without AP, respectively (RR 0.35, 95% CI 0.28 to 0.45). Sepsis occurred in 0.7% (9/1,229) in the AP group and in 4.4% (33/750) without AP (RR 0.23, 95% CI 0.12 to 0.45) (7). All these meta-analyses indicate clearly that AP is superior compared to no AP for patient relevant clinical outcomes (e.g. UTI, fever, sepsis) as well as for laboratory outcomes (e.g. bacteriuria, bacteraemia) (5-7).

The relationship of bacteriuria and UTIs varies: In the 24-hour period after TURP, many cases of bacteriuria are asymptomatic and transient, whereas bacteriuria at later periods correlates closely with clinical symptoms of infection. Therefore, bacteriuria is a widely accepted proxy for UTI incidence, particularly beyond postoperative day 2 (7). This is also shown by Wagenlehner et al. in a prospective randomised multicentre trial published in 2005 showing that 4 weeks after the operation postoperative complications (defined as UTI, sepsis, fever and shivering) in patients with bacteriuria were more frequent than in non-bacteriuric patients (18/105; 17% vs. 21/271; 8%; p < 0.01) (8).

Henceforth, to reduce postoperative UTIs after TURP, a single-dose AP is recommended by the EAU guidelines (4). But so far, for GL – a similar non-invasive intervention for subvesical obstructions – there are – to our knowledge – no international guideline recommendations for AP.

In GL, longer AP for up to 14 days has been used years ago, when the surgical procedures in use at that time resulted in more necrotic tissue in the prostatic fossa (9), causing frequent postoperative UTIs (9-12). There is no clear evidence about the frequency of UTIs under current GL surgical procedures, which have substantially improved over time. Since its clinical introduction in 1988, the GL technique has rapidly evolved and today leads to less necrotic tissue. In addition, compared to the old procedures, the 180-Watt laser today results in shorter operating- and catheterisation-duration, which are also common reasons for UTIs (12). Therefore, based on these historical data, our local in-house guidelines still recommend 6 days of AP in GL (13).

We conducted a retrospective data analysis at two of the five planned study sites from April 2016 until March 2017 including 182 patients receiving TURP and 101 GL patients (data not published): 14.3% and 13.9% were excluded due to preoperative catheterisation, bacteriuria or ongoing antimicrobial therapy (resulting in a total of 156 TURP and 87 GL included patients). In TURP, a single-dose AP was applied in 46.2% (72/156), in 53.8% (84/156) AP was extended for mainly 3 days. In GL, a single-dose AP was given in only 3.4% (3/87); 96.6% (84/87) of the patients received AP for mainly 3 days. Either during the hospitalisation (until ± 3 days) or at the follow-up (day 30; ±5 days) symptomatic UTI (based on clinical diagnosis) treated with antibiotics occurred in 9.1% (22/243) of patient (TURP in 8.3% (13/156) and 10.3% in GL (9/87)). In patients who were operated using TURP, such complications occurred in 8.3% (6/72) when they received a single-dose and also 8.3% (7/84) when they received extended AP. In GL after a single-dose AP no patient showed an infection (0/3) and 10.7% (9/84) of the infectious complications occurred after an extended AP. Overall, these observational data indicate no clear benefit of using longer AP beyond a single-dose, neither in patients with TURP nor GL.

Despite clear recommendations on AP in TURP, usual clinical care is often not in line with international guidelines: In 2013, a retrospective study analysing AP regimes in urology clinics (in 60 countries) showed significant differences among regions – in terms of treatment duration and selected substances. In clinical routine, AP was not always consistent with evidence-based guidelines (14). Local differences in the use of antimicrobial regimes may relate to the specific epidemiology of pathogens and their associated resistance pattern; however, the disagreeing duration of AP cannot be explained fully by the corresponding resistance patterns.

To assess the AP prescribing patterns in the German speaking area of Central Europe, we conducted a preliminary survey-based study among urologists in Germany, Austria and Switzerland. We asked about the application of AP and also aimed to differentiate current AP strategies in patients undergoing TURP and GL (the survey included 374 urologists, of whom 76% performed TURP and 16% GL) (15). For TURP 60% and for GL 65%, stated to routinely extended AP after surgery (i.e. longer than a single-dose AP). The majority (41% for TURP and 36% for GL) of participating urologists stated to extend AP until day 3 which is in general the day after catheter removal. 42% of urologists who use TURP and 33% who operate with GL reported to use routine preoperative AP, and 43% and 52% reported to prescribe non-recommended perioperative AP regimens (defined as substances other than TMP/SMX with or without an amino-penicillin or a cephalosporin) (15). This shows a frequent deviation from the guideline recommendations of the EAU in usual clinical care.

Antimicrobial resistance is particularly prevalent among the main pathogens of the urogenital tract (16, 17). Isolates from urological patients show high antimicrobial resistance rates. This has been explained by the frequent and extended use of antimicrobial agents partly for AP in standard urological procedures (18). Resistance rates have been increasing in parallel with the use of antimicrobials (19). Thus, clear guidelines for AP in urological interventions are essential to preserve the effectiveness of standard antimicrobial agents. These guidelines should ensure that AP is reduced to a minimum without increasing the postoperative complications for individual patients.

Therefore, in this multicentre RCT, we investigate, in patients undergoing either TURP or GL for obstructive voiding disorders, the non-inferiority of a single-dose AP with Cotrimoxazole against a 3-day AP with Cotrimoxazole in terms of the proportion of UTIs within 30 days, which require antimicrobial treatment.

## Investigational Product and Indication

Perioperative AP with two ampoules of TMP/SMX 400/80 mg solved in 250 ml sodium chloride short infusion (open label, medicinal product with marketing authorisation: Bactrim forte^®^ Infusionskonzentrat). TMP/SMX is usual care in AP and is recommended by international guidelines (i.e. EAU) as AP for TURP (18). This combination product of TMP/SMX is called Cotrimoxazole.

After the randomisation patients will receive an oral placebo tablet (lactose tablets) at the evening of the surgery, the next day and the day thereafter twice daily after breakfast and dinner, respectively (i.e. five tablets within three consecutive days, beginning at the day of surgery).

## Preclinical Evidence

There is no preclinical evidence on the question about the duration of AP. AP is in use since decades and recommended by the EAU (15). However, our survey (15) clearly indicated that despite of these recommendation, a 3-day AP is usual care in Switzerland, Germany and Austria.

## Clinical Evidence to Date

The above mentioned meta-analyses (5-7) underline the superiority of various AP strategies in terms of duration and choice of substance versus placebo to reduce postoperative bacteriuria and sepsis. Berry and Barratt conducted in their review also a sub-group analysis describing the decrease in bacteriuria with a short course AP (i.e. more than one dose administered within 72 hours) or with a single-dose AP compared to no AP (7). A short course AP decreased the incidence of bacteriuria by 72% (95% CI 42% to 87%), while a single-dose AP resulted in a decrease by 57% (95% CI 41% to 68%).

While studies have shown that adherence to AP guidelines results in health care cost savings (20), a recently published study demonstrated that strict adherence to EAU guidelines between January 2011 and December 2013 reduced antimicrobial resistance rates compared to the period before the adherence to EAU guidelines (January 2008 to December 2010) by lowering the total antimicrobial consumption (21). Furthermore, the adherence to guidelines did not increase the number of postoperative infections (before implementation of EAU guidelines: 117 of 2619 operations (4.5%); after implementation of EAU guidelines: 180 of 3529 operations (5.1%; p=0.27) (24). However, this study included all surgical urological procedures. Furthermore, the study was not implemented to investigate non-inferiority and the before-after comparison is prone to bias (e.g. surgical procedures might have improved over time).

In a systematic literature search in Medline (via PubMed, last search 20. March, 2018; detailed search strategy listed in the appendix ‘17.1 Search strategy’), we identified eight RCTs which assessed the efficacy of single-dose AP compared to prolonged AP. A total of five RCTs evaluated the same antibiotic compound as a single-dose compared to a prolonged AP (2-4 days or until catheter removal). The largest study was conducted by Hargreave et al. between 1987 and 1989 in nine European centres (22). Patients were randomised to receive single-dose ceftazidime (n=257), continuing daily ceftazidime until catheter removal (n=264) or no AP at all (n=274). The study was designed so that a difference of 9% in UTI could be detected between the non-AP group and the two AP groups. UTIs were observed significantly more frequent in patients who received no AP (33.9%; 83/245) compared to single-dose AP (18.7%; 45/240) or continuous AP (11.6%; 29/250). Furthermore, single-day AP resulted in significantly more UTIs compared to continuous AP. Another study which was published in 1984 randomised patients to receive either a single-dose of cefotaxime (1g) or to 48 hours cefotaxime regime (500 mg every 12 hours) (23). From the 106 patients who received a single-dose, 47 had at least one complication while in the 48 hours’ regime only 24 of 97 patients had any complications. It remained unclear how complications were assessed. In another RCT published in Japanese, Tsugawa and colleagues randomised patients to either a single-dose cefazoline (n=92) or a 3-day course cefazoline (n=96) (24). No difference in fever incidence (1 day AP: 3.3%; 3 days AP: 4.2%) or mean days until normalisation of urine analysis (1 day AP: 68.4 days; 3 days 68.6 days) were observed. Two RCTs conducted by Hall et al. (25) and Costa (26) had relatively small sample sizes. Hall and colleagues randomised patients to either an oral dose of fleroxacin (n=28), a single intravenous (IV) dose of fleroxacin (n=29) or an initial IV dose of fleroxacin followed by daily oral fleroxacin until catheter removal, but not more than five days. No difference in the rate of UTI (including urosepsis and fever) was observed among treatment arms (7.1% (2/28), 6.8% (2/29) and 7.4% (2/27)) in the first 6 weeks after surgery (25). In the other RCT, men received a single perioperative dose of lomefloxacin (n=20) or perioperative lomefloxacin plus daily lomefloxacin thereafter for 3 days (26). A total of 2 and 1 patient developed bacteriuria in the single AP and in the 3-day AP group, respectively. Adverse events (AEs) (e.g. nausea, vomiting and headache) seemed to be more common in the 3-day AP group.

Besides those 5 RCTs, we identified three others who assessed also the efficacy of different AP regimes (including different compounds).

One RCT, which used different cephalosporine antibiotics in the single-dose AP (i.e. ceftriaxone; n=67) compared to the long course AP (i.e. cephradine; n=76) found a lower incidence of UTI in the long course AP (11.9%) compared to single-dose AP (17.6%) (27). Another RCT which was conducted in 1987 by Duclos and colleagues, all patients (n=50) received cefotaxime one hour preoperatively (28). The half of those patients received randomly again cefotaxime one hour before catheter removal. Patients who received an additional AP before catheter removal had a lower incidence of fever and bacteriuria. In an RCT published already in 1982 patients received either cefotaxime only during the surgery (n=39) or during the surgery and every 8 hours until 24 hours after surgery (n=38) (29). Infection rates were low 3 days after surgery (0 in both groups), 5 to 7 days after surgery (3 in single AP group; 2 in 24 hours AP group), and 1 month after surgery (1 in each group).

In summary, the identified RCTs were all relatively old (published between 1984 and 1998) and none assessed the currently recommended drug combination TMP/SMX. Furthermore, the heterogeneity among the studies was high and several studies did not assess patient relevant outcomes.

A search on the International Clinical Trials Registry Platform (ICTRP) from the WHO (last search 20. March, 2018) was conducted to assess if there are currently ongoing RCTs which assess the impact of a single-dose AP compared to a prolonged AP for TURP and GL. One RCT was identified (CTRI/2017/09/009721) which planes to evaluate the efficacy of one day amikacin compared to 3 days in patients undergoing TURP. For this identified trial the target sample size is 334 patients and the primary outcome is the rate of bacteriuria 4 days after TURP.

For AP in TURP and GL, there is a large gap between usual clinical practice and evidence-based guidelines. We therefore aim to evaluate in patients who undergo TURP or GL if the guideline-conform single-dose AP is non-inferior to 3-day AP in regard to UTI proportion within 30 days, which requires further antibiotic treatment.

## Dose Rationale / Medical Device: Rationale for the intended purpose in study (pre-market MD)

A single-dose of TMX/SMX is recommended for TURP, according to the EAU guidelines (18). However, our survey among urologists in Switzerland, Germany and Austria indicated that a prolonged AP (approximately 3 days) is the current usual AP in these countries (15).

## Explanation for choice of comparator (or placebo)

Although TMP/SMX is usually recommended as single-dose AP in TURP (18), in clinic routine various antimicrobial regimes are given as AP (14, 31). Our own survey among urologists in Switzerland, Germany, and Austria has shown that more than 60% of the participating urologists extended AP. AP until day 3/day after catheter removal was the predominantly chosen regime in 41% (15). Therefore, also in the comparator group (Group B) patients receive two ampoules of TMP/SMX 400/80 mg solved in 250 ml sodium chloride short infusion during the operation, followed by a 3-day AP with Cotrimoxazole (Nopil forte^®^).

## Risks / Benefits

Based on the current evidence summarised above and the improved operation techniques, we hypothesise that a shorter AP duration (single-dose, as recommended for TURP by the EAU guidelines) does not result in more complications for the patients (see also Section “11.2 Determination of Sample Size” for detailed estimations).

Intervention or control have both shown good tolerability in clinical routine already. TMP/SMX (i.e. Cotrimoxazole) is a routinely used antimicrobial substance recommended in international and in-house guidelines as AP in TURP and UTI treatment, which is well tolerable and effective. AEs due to study medication should potentially be less common in the intervention group due to the lower exposure (single-dose vs 3-day course). Main AEs are skin reactions and gastrointestinal disorders according to the Swissmedic package leaflet. Patients with a known allergy or contraindications against TMP/SMX are excluded from the study participation. In case of such a severe reaction to TMP/SMX, the administration of study medication will be stopped immediately and necessary treatment of the allergic reaction will be performed. Continuation of the surgery and application of another AP is decided by the surgeon and the anaesthetist. The placebo contains small amounts of lactose. In case of a known intolerance, patients are excluded from the study. In case of AEs, the administration will be stopped and necessary treatment of the reaction will be performed.

## Justification of choice of study population

Antimicrobial resistance rates in urology are known to be higher than in any other clinical departments due to the overuse of antimicrobial agents for prophylactic and therapeutic indications. TURP is one of the most frequently performed urological surgeries – with almost 10,000 interventions performed in Switzerland annually (15). With most of the TURP surgeries being elective, infectious complications should be avoided while at the same time the use of antimicrobial agents should be limited to a necessary minimum. Therefore, at the five study sites, adult patients with written informed consent undergoing TURP or GL for obstructive voiding disorders are enrolled to assess if AP can be reduced to a single-dose without increasing the complications for the patients.

# STUDY OBJECTIVES

## Overall Objective

To evaluate a single-dose AP to a 3-day AP with TMP/SMX (i.e. Cotrimoxazole) in patients undergoing TURP or GL in a pragmatic, randomised controlled trial in 5 large study centres in Switzerland.

## Primary Objective

To evaluate whether a guideline-conform single-dose AP (Group A) in patients undergoing TURP or GL is non-inferior to a 3-day AP (Group B) in terms of symptomatic UTIs (based on clinical diagnosis) needing additional treatment with antimicrobial agents within 30 days after randomisation.

## Secondary Objectives

To evaluate the effect of a single-dose TMP/SMX AP compared to a 3-day AP with TMP/SMX on:

1. Symptomatic UTI (based on clinical diagnosis and judgement of the treating physician supported by measured bacteriuria of ≥10^5^ cfu/ml) treated with antimicrobial agents (**key secondary outcome**)
2. Symptomatic cystitis (based on clinical diagnosis)
3. Symptomatic epididymitis (based on clinical diagnosis)
4. Symptomatic pyelonephritis (based on clinical diagnosis)
5. Symptomatic prostatitis (based on clinical diagnosis)
6. Symptomatic urethritis (based on clinical diagnosis)
7. Urosepsis (based on clinical diagnosis)
8. Prescription of antibiotics (for any reason)
9. Asymptomatic bacteriuria of ≥10^5^ cfu/ml treated with antimicrobial agents
10. Detection of multidrug-resistant bacteria in urine culture (3MRGN, 4MRGN)
11. Any *Clostridium difficile*-associated infection
12. Re-hospitalisation (within 30 days after randomisation)
13. All-cause mortality
14. Duration of catheterisation (cumulative sum of days between randomisation and end of catheterisation or day 30)
15. Duration of hospital stay (cumulative sum of hospital days between randomisation and day 30)
16. Duration of intensive care unit stay (cumulative sum of ICU days between randomisation and day 30)
17. Prescribed defined daily doses (DDD) of antibiotics (cumulative sum of DDD from randomisation to day 30)
18. Change of International Prostate Symptom Score (prior to randomisation and at day 30 after randomisation)
19. Change of Quality of life Score (prior to randomisation and at day 30 after randomisation)

Within 30 days after randomisation (if not stated otherwise)

## Safety Objectives

To evaluate the impact of a single-dose AP in comparison to a 3-day AP, in patients undergoing TURP or GL, on AEs and serious adverse events (SAEs).

# STUDY OUTCOMES

All outcomes are events within 30 days (±5 days) after randomisation if not otherwise stated.

## Primary Outcome

Symptomatic UTI (based on clinical diagnosis) treated with antimicrobial agents (as per clinical judgement of the treating physician)

## Secondary Outcomes

1. Symptomatic UTI (based on clinical diagnosis and judgement of the treating physician supported by measured bacteriuria of ≥10^5^ cfu/ml) treated with antimicrobial agents (**key secondary outcome**)
2. Symptomatic cystitis (based on clinical diagnosis)
3. Symptomatic epididymitis (based on clinical diagnosis)
4. Symptomatic pyelonephritis (based on clinical diagnosis)
5. Symptomatic prostatitis (based on clinical diagnosis)
6. Symptomatic urethritis (based on clinical diagnosis)
7. Urosepsis (based on clinical diagnosis)
8. Prescription of antibiotics (for any reason)
9. Asymptomatic bacteriuria of ≥10^5^ cfu/ml treated with antimicrobial agents
10. Detection of multidrug-resistant bacteria in urine culture (3MRGN, 4MRGN)
11. Any *Clostridium difficile*-associated infection
12. Re-hospitalisation (within 30 days after randomisation)
13. All-cause mortality
14. Duration of catheterisation (cumulative sum of days between randomisation and end of catheterisation or day 30)
15. Duration of hospital stay (cumulative sum of hospital days between randomisation and day 30)
16. Duration of intensive care unit stay (cumulative sum of ICU days between randomisation and day 30)
17. Prescribed defined daily doses (DDD) of antibiotics (cumulative sum of DDD from randomisation to day 30)
18. Change of International Prostate Symptom Score (prior to surgery and at day 30 after randomisation)
19. Change of Quality of life Score (prior to surgery and at day 30 after randomisation)

All sensitivity and subgroup analyses will be done for the primary outcome and for the key secondary outcome (see Section 11).

## Other Outcomes of Interest

None.

## Safety Outcomes

- AEs
- SAEs

All other relevant ‘safety outcomes’ (i.e. complications) are assessed as primary or secondary outcomes.

# STUDY DESIGN

## General study design and justification of design

This is a randomised controlled, non-inferiority, parallel group, double-blinded trial with a 1:1 randomisation ratio in five urological departments in Switzerland. Study participations are either randomised to a guideline-conform single-dose AP (Group A) or to a 3-day AP (Group B). Physicians, patients and outcome assessors will be blinded. The trial is based on routinely collected data during TURP and GL of the prostate until day 30 after surgery (i.e. randomisation).

Only a randomised controlled trial would be able to provide reliable evidence to guide decision-making in this setting. Non-randomised observational methods would carry a high risk of bias and not allow to make causal inferences about the comparative merits of both treatment strategies.

## Methods of minimising bias

### 6.2.1 Randomisation

Patients will be randomly allocated to both groups in a 1:1 ratio. Randomisation will be performed via the electronic data capture (EDC) system, which is accessible via a standard browser on a WWW-connected device. The investigators will enter patient details into the electronic case report form (eCRF) via a secure web interface before randomisation takes place. In case of technical problems not allowing randomisation using the web-based randomisation system, local investigators will use the study treatment pack with the lowest number available in the centre and communicate the used study treatment pack to the trial management group. The used study treatment pack will then be assigned to the patient via the web-based eCRF.

The randomisation procedure will include a variance minimisation algorithm which will ensure that the study arms are balanced for some potential confounder variables, specifically centre and surgery type (TURP or GL). In order to avoid predictable alteration of treatment allocation, and thus potential loss of allocation concealment, patients will be allocated with a probability of 0.80 to each treatment group that would minimise the difference between the groups on the key prognostic factor.

### 6.2.2 Blinding procedures

The tailoring and randomisation of the study medication will be performed by the Hospital Pharmacy of the University Hospital Basel according to Good Manufacturing Practice. Perioperative AP will be two intravenous ampoules of TMP/SMX 400/80 mg (Bactrim Inf Konz^®^) solved in 250 ml sodium chloride short infusion in both groups. Each study medication package consists of either five tablets of placebo (Group A) or five tablets of TMP/SMX (Nopil forte^®^) 800/160mg (Group B) using licensed product repacked in a new immediate container which is blinded.

The placebo tablets are optically similar, but not identical to the TMP/SMX (Nopil forte^®^) 800/160mg. The blinding is ensured via non-transparent immediate containers. Perfectly identical placebos were not available.

After randomisation, the study team will provide the corresponding medication number to a nurse who is responsible for drug distribution. This nurse will bring the study medication to the patient and instruct the patient when to take the tablets.

Patients will then take the study drug independently according to the instruction of the nurse and an information sheet handed out with the pill container. The nurse will regularly ask if the study drug was taken and will document this information in the hospital information system. On day 3, the study nurse will acoustically check whether the container is empty. Drug containers which are not empty will be sent back to the Hospital Pharmacy of the University Hospital Basel, where the remaining tablets will be counted (the information will be recorded in the eCRF) and destroyed.

Evaluation of the primary outcome parameter will be done by an independent and blinded outcome assessor.

### 6.2.3 Other methods of minimising bias

Not applicable.

## Unblinding Procedures (Code break)

If an unblinding due to safety concerns is required, local investigators and the delegated study personnel can decide for unblinding.

Otherwise the EDC software secuTrial^®^ allow for unblinding by selected users. Each unblinding will be documented.

Unblinding by opening the re-sealed study medication is prohibited but the possibility to do so cannot be ruled out.

# STUDY POPULATION

This is a multicentre study at 3 academic hospitals, 1 community and 1 private hospital in Switzerland. All patients scheduled for TURP or GL due to voiding disorders (e.g. benign prostate hyperplasia, obstructive prostate cancer) at the 5 study sites meeting the eligibility criteria will be asked to participate. Informed consent will take place at the outpatient clinic, when the surgery is planned.

## Eligibility criteria

#### Inclusion criteria

- Adult male patients (≥18 years)
- Obstructive voiding disorder (e.g. benign prostate hyperplasia, obstructive prostate cancer)
- Planned TURP or GL

#### Exclusion criteria

- Evidence for (catheter associated-) UTI, with or without antibiotic treatment in the last 7 days prior to randomisation.
- Any evidence of a history of positive urine culture (cfu ≥10^5^/ml in midstream-urine with no more than two species) and resistance to TMP/SMX in the last 7 days prior to randomisation (32).
- Known contraindication against study drugs according to the Swissmedic package leaflet (e.g. known liver dysfunction, renal insufficiency; patients with glomerular filtration rate (calculated by the MDRD or CKD-EPI) <30ml/min or dialysis patients will be excluded).
- Antibiotic treatment for any reason within 7 days prior to randomisation
- Indication for AP for other reasons (e.g. endocarditis prophylaxis, transplanted patients under systemic immunosuppression).

## Recruitment and screening

At each of the 5 study sites, a study nurse will identify potentially eligible patients by checking the reason for consultation (e.g. preoperative consultations) in the hospital information system. The physician who is doing the informed consent talk for the surgery (approximately 1 week before surgery) will inform the patients about the study, including all relevant risks and benefits, as well as that participation is voluntary and that withdrawal is possible any time without mentioning any reason. With consenting to take part in the study, the patient agrees that routinely collected data before surgery and until day 30 (±5 days) after randomisation can be used anonymised even if the patient stops the study medication or drops out of the study. However, the patient may also withdraw his consent to data usage at any time without mentioning any reason. After explanation of all study details, written consent from participants will be obtained. Participants will not receive any financial compensation for participation.

In 2016, a total of 869 patients underwent either TURP or GL at the 5 study centres. Of those patients approximately 85% (738) would have been eligible for this trial. We assume that approximately 80% (590) of those patients would be willing to participate. Therefore, we assume that approximately 470 patients can be recruited each year and that the recruitment should be finished after a total of 3.5 years (i.e. October 2018 to March 2022). In case of unanticipated difficulties with recruitment, additional study sites in Switzerland could be added.

## Assignment to study groups

Patients will be automatically assigned to study groups in a 1:1 ratio using a web-based randomisation tool.

## Criteria for withdrawal / discontinuation of participants

Enrolled patients have always the opportunity to withdraw from the trial without mentioning any specific reason at any time. In case of a SAE, the study drug will be stopped immediately and necessary treatment will be performed. In case of any complications, which require an antimicrobial therapy, the study drug will be stopped. Participations who stopped the study drug or withdrew consent will be asked if they are anyway willing to conduct the routine follow-up visit 30 days after surgery. Patients can state that they do not wish to be contacted for the study anymore and that also their physician shall not be contacted for outcome assessment.

# STUDY INTERVENTION

## Identity of Investigational Products

TMP/SMX 400/80 mg (Bactrim Inf Konz^®^) and TMP/SMX 800/160mg (Nopil forte^®^ tablets) will be purchased from Roche Pharma AG and Mepha Pharma AG, respectively. Placebo 500 mg tablets will be ordered from Fagron GmbH & Co. KG.

### 8.1.1 Experimental Intervention (treatment / medical device)

Group A: The intervention is a guideline-conform single-dose AP with TMP/SMX. Perioperative AP will be two intravenous ampoules of TMP/SMX (Bactrim Inf Konz^®^) 400/80 mg solved in 250 ml sodium chloride short infusion. After surgery patients receive an oral placebo on the evening of the surgery and thereafter twice daily on day 1 and 2 after randomisation (after breakfast and dinner).

### 8.1.2 Control Intervention (standard/routine/comparator treatment / medical device)

Group B: The control group represents the usual clinical care (3 days of AP) and receives two ampoules of TMP/SMX (Bactrim Inf Konz^®^) 400/80 mg solved in 250 ml sodium chloride short infusion during surgery and additionally oral TMP/SMX (Nopil forte^®^) 800/160mg on the evening of the surgery and thereafter twice daily on day 1 and 2 after randomisation (after breakfast and dinner).

### 8.1.3 Packaging, Labelling and Supply (re-supply)

The pharmacy of the University Hospital Basel will repack, label, and dispense the oral study medication (i.e. oral TMP/SMX 800/160mg (Nopil forte^®^) or placebo tablets according to the randomisation scheme). Study medication will be packed into non-transparent containers. The personnel who will conduct the labelling and packing will have no further role in this study. Two intravenous ampoules of TMP/SMX 400/80 mg solved in 250 ml sodium chloride short infusion (Bactrim Inf Konz^®^) is usual care and in both treatment groups identical. Therefore, intravenous TMP/SMX (Bactrim Inf Konz^®^) will be provided directly from the study centres.

### 8.1.4 Storage Conditions

The study medication are stored at the respective medicine cabinets of the participating study sites according to the notifications of the Swissmedic package leaflet. Intravenous TMP/SMX and TMP/SMX tablets are stored at room temperature (15-30 °C) and only substances with valid expiry date are being used. Prepared infusion solution is used within six hours after preparation.

## Administration of experimental and control interventions

### 8.2.1 Experimental Intervention

At the day of surgery, the anaesthetists (not related to the study) will be informed via the hospital information system and the study nurse that the patient is included in the study and that TMP/SMX (two ampoules of TMP/SMX 400/80 mg (Bactrim Inf Konz^®^) solved in 250 ml sodium chloride short infusion) has to be used as an AP.

After surgery, the nurses will be informed over the hospital information system that the patient needs to receive the oral study medication. They will bring the container with the oral study medication and instruct the patient when to take it (i.e. one oral tablet at the evening of the surgery, the next day and the day thereafter twice daily after breakfast and dinner, respectively (i.e. five tablets within three consecutive days, beginning at the day of surgery). An information sheet with the dosage instruction is handed out as well.

### 8.2.2 Control Intervention

See section 8.2.1.

## Dose / Device modifications

No dose modifications are planned.

## Compliance with study intervention

The patients will independently take the study medications at day 0 (evening), day 1 and 2. In routine care, nurses regularly ask about the intake and enter this into the hospital information system. The study nurse will control the drug intake by confirming that the corresponding drug containers are empty 3 days after randomisation. To remain blinded the confirmation will be carried out by acoustically checking the drug container (shaking). In case that the drug containers were not emptied, the container will be sent to the Hospital Pharmacy of the University Hospital Basel, where the remaining tablets will be counted and destroyed.

## Data Collection and Follow-up for withdrawn participants

Patients who withdraw from the study will be invited to conduct the routine control on day 30 after randomisation. Data which is raised until withdrawal will be analysed. Withdrawn patients have also the right and the possibility to explicitly state that the data from the routine control on day 30 shall not be included into the eCRF. If they do wish that their data is deleted after the final analysis, withdrawn patients can request that their data will be deleted from the eCRF upon request of investigators by a data manager from the clinical trial unit (CTU) Basel. Withdrawn patients will not be replaced.

The majority of the data is routinely collected and is saved in the hospital information system.

## Trial specific preventive measures

If an antibiotic has to be administered during the first 3 days after randomisation, the study medication needs to be stopped immediately.

## Concomitant Interventions (treatments)

Concomitant interventions are allowed whenever necessary as in usual care based on the clinical judgment of the physician. All concomitant drug interventions of special interest at baseline and within the first three days as well as at follow-up will be recorded in the eCRF. Concomitant interventions of special interest which patients take regularly at baseline are non-steroidal anti-inflammatory drugs (NSAID), anticoagulation, antiplatelet drugs and immunosuppressive drugs (e.g. steroids). Concomitant care of special interest within the first three days after randomisation and at follow-up are NSAIDs.

## Study Drug / Medical Device Accountability

The oral study medication will be delivered from the Pharmacy of the University of Basel to the local investigators from the participating study centres. The local investigators are responsible for adequate storage. Since perioperative intravenous AP with single-dose TMX/SMX is provided in both groups, the local investigators are responsible to provide the intravenous study drug. Nurses will remind the patients about intake of each oral tablet.

## Return or Destruction of Study Drug / Medical Device

All study drugs will be administered during the hospital stay. Unused study drugs will be destroyed by the Hospital Pharmacy of the University hospital Basel.

# STUDY ASSESSMENTS

## Table of study procedures and assessments

|  | **STUDY PERIOD** | | | | | |
| --- | --- | --- | --- | --- | --- | --- |
|  | **Enrolment** | **Randomisation** | **Post-randomisation** | | | **Close-out** |
| **TIMEPOINT** | **Approximately -7 days (±)** | **Day 0** *(operation)* | **Day 1 and 2**  *(hospital stay)* | **Day 3**  *(hospital discharge)* | ***Unscheduled Visits*** *(between hospital discharge and close-out visit)* | **Day 30 (±5 days)**  *(follow-up visit)* |
| ENROLMENT:  Eligibility screen  Informed consent  Allocation |  |  |  |  |  |  |
|  | X |  |  |  |  |  |
|  | X |  |  |  |  |  |
|  |  | X |  |  |  |  |
| INTERVENTIONS: |  |  |  |  |  |  |
| Group A: single-dose AP |  | X | X |  |  |  |
| Group B: 3-day AP |  | X | X |  |  |  |
| ASSESSMENTS: |  |  |  |  |  |  |
| Baseline variables   - Study site - Study ID - BMI - Indication for operation - ASA score - Blood counts - PSA - Prostate volume, post voiding residual volume - catheterisation - International Prostate Symptom Score/Quality of Life score - History of urinary tract infections | X |  |  |  |  |  |
| Surgery variables:   - Method of operation - Qualification of surgeon - Operating time - Time of AP - Change of AP - Intraoperative complication - Postoperative catheter - Postoperative catheter material |  | X |  |  |  |  |
| Outcome variables: |  |  |  |  |  |  |
| **Primary outcome**: Symptomatic UTI (based on clinical diagnosis) treated with antimicrobial agent (as per clinical judgement of the treating physician) |  |  |  |  |  | X |
| a) Symptomatic UTI (based on clinical diagnosis and judgement of the treating physician supported by measured bacteriuria of ≥10^5^ cfu/ml) treated with antimicrobial agent (key secondary outcome) |  |  |  | X | X | X |
| b-f) Symptomatic cystitis, epididymitis, pyelonephritis, prostatitis, urethritis (all based on clinical diagnosis) |  |  |  |  |  | X |
| g) Urosepsis (based on clinical diagnosis) |  |  |  |  |  | X |
| 1. Prescription of antibiotics (for any reason) |  |  |  |  |  | X |
| 1. Asymptomatic bacteriuria of ≥10^5^ cfu/ml treated with antimicrobial agents |  |  |  | X | X | X |
| 1. Detection of multidrug-resistant bacteria in urine culture (3MRGN, 4MRGN) |  |  |  | X | X | X |
| 1. Any *Clostridium difficile*-associated infection |  |  |  |  |  | X |
| 1. Re-Hospitalisation (within 30 days after randomisation) |  |  |  |  |  | X |
| 1. All-cause mortality |  |  |  |  |  | X |
| 1. Duration of catheterisation (cumulative sum of days between randomisation and end of catheterisation or day 30) |  |  |  |  |  | X |
| 1. Duration of hospital stay (cumulative sum of days between randomisation and discharge or day 30) |  |  |  |  |  | X |
| 1. Duration of intensive care unit stay (cumulative sum of days between randomisation and discharge from ICU or day 30) |  |  |  |  |  | X |
| 1. Prescribed defined daily doses (DDD) of antibiotics (cumulative sum of DDD from randomisation to day 30) |  |  |  |  |  | X |
| 1. Change of International Prostate Symptom Score (prior to randomisation and at day 30 after randomisation) | X |  |  |  |  | X |
| 1. Change of Quality of life Score (prior to randomisation and at day 30 after randomisation) | X |  |  |  |  | X |
| Adverse events |  |  |  | X |  |  |
| Serious adverse events |  | X | X | X | X | X |
| Concomitant care | X |  |  | X |  | X |

Abbreviations: AP, antimicrobial prophylaxis; ASA, American Society of Anesthesiologists; BMI, body mass index; cfu, colony-forming units; DDD, defined daily doses; ID, identification (number); ICU, intensive care unit; PSA, prostate specific antigen; UTI, urinary tract infection.

## Assessments of outcomes

The vast majority of outcomes will be assessed within routine follow-up (See also section “9.1 Table of study procedures and assessments”) using routinely collected data from the hospital information system.

Additionally, patients will be asked at the 30 day follow-up visit if any symptoms occurred since the hospital discharge, if symptoms were potentially related to an UTI (i.e. flank pain, suprapubic pain, urgency, frequency, fever), if a physician was consulted, and if antibiotics were taken. All these information will be confirmed by contacting the treating physician.

We generally assume that the hospital information system at each study site provides highly accurate information. However, we will evaluate the agreement of data captured via the hospital information system and actively collected data (for example by confirmation by the treating physician) to understand the accuracy and reliability of this data. In case that there is any disagreement between the data sources, the physician who conducted the 30 day follow-up (or in case of no appearance, who was supposed to conduct the 30 day follow-up) will document and resolve these discrepancies.

### 9.2.1 Assessment of primary outcome

The primary outcome will be assessed at the routine control 30 days after randomisation. The following procedure will be followed:

(1) Before the patient arrives, the clinician who will perform the routine control or the study nurse will consult the hospital information system and assess if the primary endpoint occurred during the hospital stay or if the patient was re-hospitalised due to a ‘Symptomatic UTI (based on clinical diagnosis) treated with antimicrobial agents’.

(2) During the routine control, the clinician will check if the primary endpoint is currently present and additionally the patient will be asked if he received any antibiotics or had a diagnosis of an UTI between hospital discharge and the routine control.

(3) In case that the patient reports that either an antibiotic was taken or such diagnosis was made, (and also if any uncertainty about the non-occurrence of the primary endpoint exists), the following clinician will be contacted:

a) the treating physician (typically the patient’s general practitioner)

b) or the corresponding physician who prescribed the antibiotics or made the diagnosis to confirm the primary endpoint.

In case the patient misses the follow-up routine control at 30 days, the routine control will be re-scheduled (the date must not be longer than 35 days after randomisation). If this is not possible, information about the same procedure as above (1 to 3) is followed, but the patient is called via phone and asked about the occurrence of the primary endpoint.

### 9.2.2 Assessment of secondary outcomes

**Symptomatic UTI (based on clinical diagnosis supported by measured bacteriuria of ≥10^5^ cfu/ml) treated with antimicrobial agents (key secondary outcome)**

The key secondary outcome will be assessed in the same way as the primary outcome (see section “9.2.1 Assessment of primary outcome”) within the same procedure and from the same persons to be contacted. Whenever the primary outcome occurred, it will be assessed in the hospital system and in the contacting of the physicians, if this event was confirmed by a bacteriuria of ≥10^5^ cfu/ml. Urine cultures are performed at day 3 and day 30 as well during unscheduled visits. Urine culture results can be found in the hospital information system. From all urine samples it will be recorded in the eCRF how they were collected (i.e. midstream, from catheter). We will consider possible between-group differences in this regard in the interpretation of this outcome.

**Symptomatic cystitis, epididymitis, pyelonephritis, prostatitis, urethritis, urosepsis (based on clinical diagnosis)**

The same procedure as for the primary endpoint will be followed (see section “9.2.1 Assessment of primary outcome”; 1) and 2)). In case that the patient reports that either an antibiotic was taken or an UTI diagnosis was made, (and also if any uncertainty about the non-occurrence of the primary endpoint exists), the following clinician will be contacted and asked about the occurrence of cystitis, epididymitis, pyelonephritis, prostatitis, urethritis, urosepsis (based on clinical diagnosis):

a) the treating physician (typically the patient’s general practitioner)

b) or the corresponding physician who made the diagnosis to confirm the primary endpoint.

**Prescription of antibiotics (for any reason)**

This secondary outcome will be assessed similar like the primary outcome (see section “9.2.1 Assessment of primary outcome”) at day 30. All antibiotic prescriptions will be counted independent if a symptomatic UTI occurred. The reason of antibiotic prescription will be recorded in the eCRF.

**Prescribed defined daily doses (DDD) of antibiotics (cumulative sum of DDD from randomisation to day 30)**

From each prescribed antibiotic (assessment same as for primary outcome; see section “9.2.1 Assessment of primary outcome”) the following information will be recorded: Specific compound which, dose in mg per intake, how many intakes per day, and for how many days the antibiotic was prescribed. Based on this information the defined daily doses according to the ATC system (33) will be calculated. If a patient states that the prescribed antibiotics were not taken, these will anyway be counted under “prescribed DDD”.

**Asymptomatic bacteriuria of ≥10^5^ cfu/ml treated with antibiotics**

This outcome will be assessed based on the information from the previous listed outcomes. Events will be counted if patient received antibiotics based on a bacteriuria ≥10^5^ cfu/ml without any symptomatic signs for a UTI.

**Detection of multidrug-resistant bacteria in urine culture**

Routine urine culture and resistance patterns are performed prior to the surgery, at day 3 and day 30. Urine culture are found in the hospital information system.

1. Before the patient arrives the clinician who will perform the routine control or the study nurse will consult the hospital information system and assess the urine culture result and the resistance pattern.
2. Depending on these results 3MRGN and 4MRGN is assessed according to the definition by the Robert Koch Institute (34).
3. If a patient reported during the assessment that another physician was consulted (see section “9.2.1 Assessment of primary outcome”) the clinician will contact the corresponding to receive the corresponding urine culture results with resistance pattern. Resistance pattern and 3MRGN and 4MRFN will be assessed as described in 1) and 2).

**Any *Clostridium difficile*-associated infection**

The following procedure will be performed at day 30. First, before the patient arrives the clinician who will perform the routine control or the study nurse will consult the hospital information system and assess if the endpoint occurred during the hospital stay or if the patient was re-hospitalized due to a ‘*Clostridium difficile*-associated infection’, documented by microbiological method according to guidelines issued by the European Society for clinical microbiology & infection (ESCMID) (35). During the routine control, the clinician will check if the patient has symptoms and/or signs suggestive of a *Clostridium difficile*-associated infection. If yes, specific microbiological stool tests are performed (depending on the respective local algorithm at each study center for diagnosing a *Clostridium difficile*-associated infection).

**Duration of catheterisation (cumulative sum of catheter days between randomisation and day 30)**

At the routine control the study nurse will enter the day of catheter removal into the eCRF based on the information provided in the hospital information system. Additionally, it will be reported if subsequent catheters were used (including the date of application and removal). Based on these data the duration of catheterisation will be calculated.

**Duration of hospital stay (cumulative sum of hospital days between randomisation and day 30)**

At day 30 the study nurse will enter the discharge day after randomisation into the eCRF by consulting the hospital information system. Furthermore, the hospital information system will be consulted and the patient is asked, if between hospital discharge and routine control hospitalisation (days) occurred. In case that the patient reports that the endpoint occurred between hospital discharge and routine control (i.e. another hospital; and also if any uncertainty about the non-occurrence of the endpoint exists) the clinician will contact the corresponding physician and gets the information. The days of re-hospitalisations and the days of discharges will be reported. Based on these information the duration of hospital stay will be calculated.

**Duration of intensive care unit stay (cumulative sum of ICU days between randomisation and day 30)**

At the routine control, the study nurse calculates the total days of ICU stay by consulting the hospital information system. Furthermore, the patient is asked, if between hospital discharge and routine control, a rehospitalisation and ICU stay has occurred and for how long (days). In case that the patient reports that a rehospitalisation/ICU stay occurred between hospital discharge and routine control (i.e. at another hospital; and also if any uncertainty exists), the clinician will contact the corresponding physician and will obtain the respective information.

**Re-Hospitalisation (within 30 days after randomisation)**

See endpoint ‘Duration of hospital stay”.

**Change of International Prostate Symptom Score (prior to randomisation and at day 30 after randomisation)**

Prior to the surgery and at the routine control at day 30, patients are routinely handed out the validated International Prostate Symptom Score (36) consisting of 7 questions. Each question concerning urinary symptoms allows the patient to choose one out of six answers indicating increasing severity of the particular symptom. The answers are assigned points from 0 to 5. The total score can therefore range from 0 to 35 (asymptomatic to very symptomatic). The change of the score is calculated by subtracting the score at day 30 from the score at enrolment.

**Change of Quality of life Score (prior to randomisation and at day 30 after randomisation)**

Prior to the surgery and at the routine control at day 30, patients are routinely handed out the International Prostate Symptom Score (36, 37). This questionnaire is added by the Quality of life Score (36) consisting of one question allowing the patients to choose one out of seven answers from 0-6 (delighted – terrible). The change of the score is calculated by subtracting the score at day 30 from the score at enrolment.

**All-cause mortality**

First, before the patient arrives the clinician who will perform the routine control or the study nurse will consult the hospital information system and assess if the endpoint occurred during the hospital stay or if the patient was re-hospitalised and the endpoint occurred. In case the patient misses the follow-up routine control at 30 days, the study nurse consults the patient by phone. If the patient is not available, the treating physician (e.g. general practitioner) is called to assess the endpoint and, if applicable, the date and cause of mortality.

### 9.2.3 Assessment of other outcomes of interest

There are no further outcomes planned to be assessed.

### Assessment of safety outcomes

#### 9.2.4.1 (Serious) Adverse events

To assess any potential harms, the study team evaluates SAE according to the ICH E2A guidelines (38) as “definitely”, “probably”, “possibly”, “unlikely” and “not related”. The “Common Terminology Criteria for Adverse Events”, Version 5.0 terminology is going to be used for (S)AE occurring throughout this study: 1 – Mild (does not influence activities of daily living), 2 – Moderate (influences activities of daily living), 3 – Severe (makes some activities of daily living impossible), 4 – Life-threatening, 5 – Death. For further information please refer to Section 10.1.1. The study personnel will automatically be reminded at any study visit (planned or unplanned) from the time point of final treatment allocation to assess SAE on a separate form if any occurred. At day 3, AEs of special interests (e.g. diarrhea, nausea, vomiting, allergic reaction, and neurological disorder) will be assessed. Additionally, other not pre-specified AEs can be entered into the eCRF.

#### Laboratory parameters

Please refer to Section 9.2.1 and 9.2.2.

#### Vital signs

Please refer to Section 9.2.1 and 9.2.2.

### 9.2.5 Assessments in participants who prematurely stop the study

As the vast majority of outcomes is based on routinely collected data, outcomes can also be assessed if participants prematurely stop the study.

## Procedures at each visit

### See also Section 9.1.

### 9.3.1 Enrolment

For each patient at enrolment (approximately 7 days before surgery): eligibility screen, informed consent and assessment of baseline variables (study site, study ID, BMI, ASA score, blood counts, anticoagulation, PSA, prostate volume, post voiding residual volume, catheterisation, International Prostate Symptom Score/Quality of Life score, history of urinary tract infections) is performed.

### 9.3.2 Allocation

Before the randomisation takes place, each patient will undergo a final eligibility check based on all at that time point available evidence from the laboratory and from asking the patient (i.e. evidence for UTI, cfu ≥10^5^/ml or antibiotic treatment in the last 7 days before surgery (i.e. randomisation)). If all questions can be answered with no (i.e. no evidence for (i) UTI; (ii) cfu ≥10^5^/ml; (iii) antibiotic treatment in the last 7 days before surgery (i.e. randomisation)), the patient will be randomised and will receive an identification code number via eCRF. The assessed parameters from the surgery are listed under Section 9.1. In the evening after the operation the patients will receive a drug container with the study medication (i.e. 5 tablets). They will be instructed to swallow one tablet on the evening of day 0 and the consecutive 4 tablets on day 1 and day 2 (always one after breakfast and one after dinner). Nurses will remind the patients about the intake of each tablet. Patients get and information sheet with the dosage instruction.

### 9.3.3 Day 1 and 2

Nurses will remind the patients about the intake of each tablet.

### 9.3.4 Day 3

At day 3, a urine culture (with antimicrobial resistance pattern) is performed. Furthermore, AEs of special interest as well as other potential AEs will be assessed. The study nurse will check if the entire study medication was taken as prescribed (See section 8.4)

### 9.3.5 Unscheduled Visits

Any unscheduled visits occurred during hospital discharge will be assessed at the 30 day follow-up by consulting the hospital information system and by asking the patient.

### 9.3.6 Follow-up after 30 days

All endpoints will be assessed at day 30 (±5 days; for more details see Section 9.2.1-9.2.4). In case that a patient will not conduct the 30 day visit, the treating physician or the study nurse will try to find an alternative date within the 5 day window. If this is not possible, the requested information for the primary endpoint will be asked by phone.

# SAFETY

## Drug studies

During the entire duration of the study, all SAEs are collected, fully investigated and documented in source documents and eCRFs. Study duration encompassed the time from when the participant signs the informed consent until the last protocol-specific procedure has been completed, including a safety follow-up period planned at close-out day 30.

### 10.1.1 Definition and assessment of (serious) adverse events and other safety related events

We follow the definition of the ICH E2A guidelines (39): “An **Adverse Event (AE)** is any untoward medical occurrence in a patient or a clinical investigation participant administered a pharmaceutical product and which does not necessarily have a causal relationship with the study procedure. An AE can therefore be any unfavourable and unintended sign (including an abnormal laboratory finding), symptom, or disease temporally associated with the use of a medicinal (investigational) product, whether or not related to the medicinal (investigational) product.

A **Serious Adverse Event (SAE)** is classified as any untoward medical occurrence that:

- results in death
- is life-threatening
- results in re-hospitalisation
- results in persistent or significant disability/incapacity

In addition, important medical events that may not be immediately life-threatening or result in death, or require hospitalisation, but may jeopardise the patient or may require intervention to prevent one of the other outcomes listed above should also usually be considered serious.” (ICH E2A)

Since all patients are adult men, “congenital anomaly/birth defect” will not be applicable for this trial.

Prolonged hospitalisation is common in the population of interest and is therefore not considered as an SAE.

SAEs should be followed until resolution or stabilisation. Participants with ongoing SAEs at study termination (including safety visit) will be further followed up until recovery or until stabilisation of the disease after termination.

Assessment of Causality

Both Investigator and Sponsor/Principle Investigator make a causality assessment of the event to the study drug, based on the criteria listed in the ICH E2A guidelines:

| Relationship | Description |
| --- | --- |
| Definitely | Temporal relationship  Improvement after dechallenge*  Recurrence after rechallenge  (or other proof of drug cause) |
| Probably | Temporal relationship  Improvement after dechallenge  No other cause evident |
| Possibly | Temporal relationship  Other cause possible |
| Unlikely | Any assessable reaction that does not fulfil the above conditions |
| Not related | Causal relationship can be ruled out |
| *Improvement after dechallenge only taken into consideration, if applicable to reaction | |

Unexpected Adverse Drug Reaction

An “unexpected” adverse drug reaction is an adverse reaction, the nature or severity of which is not consistent with the applicable Summary of Product Characteristics (SmPC) of Bactrim Inf Konz^®^ and Nopil forte^®^.

Suspected Unexpected Serious Adverse Reactions (SUSARs)

The Sponsor/Principle Investigator evaluates any SAE that has been reported regarding seriousness, causality and expectedness. If the event is related to the investigational product and is both serious and unexpected, it is classified as a SUSAR. If a SUSAR is suspected, the local investigator and the principle investigator are allowed to break the blinding in the EDC system. Unblinding will be documented in the EDC system.

Assessment of Severity

The “Common Terminology Criteria for Adverse Events”, Version 5.0 terminology is going to be used for adverse events occurring throughout this study: 1 – Mild, 2 – Moderate, 3 – Severe, 4 – Life-threatening, 5 – Death (40).

### 10.1.2 Reporting of serious adverse events (SAE) and other safety related events

Reporting of SAEs

All SAEs must be reported immediately and within a maximum of 24 hours to the Sponsor/Principle Investigator of the study. The Sponsor/Principle Investigator will receive an automatic message from the EDC system in case a SAE was entered into the eCRF at any study site. The Sponsor/Principle Investigator will re-evaluates the SAE within the eCRF and confirm or adapted the filled out SAE form.

Any SAEs resulting in death are reported to the lead Ethics Committee (by the Sponsor/Principle Investigator or his/her delegated person) within 7 days via BASEC and to Swissmedic.

SUSARs will be reported to the Ethics Committee (local event via local Investigator) via BASEC and to Swissmedic (via Sponsor/Principle Investigator) within 7 days, if the event is fatal, or within 15 days (all other events).

The Sponsor/Principle Investigator must inform all investigators participating in the clinical study of the occurrence of a SUSAR. All in the trial involved Ethics Committees will be informed about SUSARs in Switzerland via Sponsor/Principle Investigator via BASEC according to the same timelines.

Reporting of Safety Signals

All suspected new risks and relevant new aspects of known adverse reactions that require safety-related measures, i.e. so called safety signals, must be reported to the Sponsor/Principle Investigator within 24 hours. The Sponsor/Principle Investigator must report the safety signals within 7 days to the Ethics Committee (local event via local Investigator) via BASEC and to Swissmedic.

The Sponsor/Principle Investigator must immediately inform all participating investigators about all safety signals. The other in the trial involved Ethics Committees will be informed about safety signals in Switzerland via the Sponsor/Principle Investigator.

Reporting and Handling of Pregnancies

Not applicable

Periodic reporting of safety

SAE which did not result in death of the person as well as all occurred deaths will be summarised in an annual safety report that is submitted once a year to the local Ethics Committees and Swissmedic via the Sponsor/Principle Investigator.

### 10.1.3 Follow-up of (Serious) Adverse Events

During the study every step is taken to ensure, that all (S)AEs are identified and documented. Treating doctors and study centers are responsible to treat the (S)AEs and follow-up (S)AEs. At the routine follow-up at day 30 it is ensured, that all (S)AEs are identified and well treated. If a (S)AE occurs, all efforts will be undertaken to treat these (S)AEs (e.g. antiallergic treatment in case of an allergic reaction) and follow-up until it is resolved is ensured.

## Medical Device Category C studies

Not applicable.

## Medical Device Category A studies

Not applicable.

# STATISTICAL METHODS

## Hypothesis

The primary objective is to evaluate whether single-dose AP with TMP/SMX is non-inferior to 3-days AP with TMP/SMX (i.e. usual clinical care) regarding event rates of symptomatic UTIs. The non-inferiority margin δ is defined as the absolute difference in the event rates under intervention (p_sdAP_) compared to control (p_3dAP_).

H_0_: p_sdAP_ - p_3dAP_ ≥ δ

H_A_: p_sdAP_ - p_3dAP_ < δ

Non-inferiority will be declared if the upper limit of the two-sided 95% confidence interval of the absolute difference in event rates, p_sdAP_ - p_3dAP_, is smaller than the non-inferiority margin δ.

## Determination of Sample Size

Sample size was estimated to be able to able to show non-inferiority of single-dose AP compared to 3-day AP with at least 80 % power (1 – *β* = 0.8) at a significance level of *α* = 5 %, applying a 1:1 randomisation scheme. A drop-out rate of 10% was considered.

Determination of the non-inferiority margin $\delta$: There is no direct data available describing the effect of 3-day AP with TMP/SMX versus placebo on our primary endpoint. Based on internal data from the involved clinics, we assumed an expected UTI rate within 30 days under usual care (3-day AP) of 0.09 (9 %). A comprehensive meta-analysis (5) reported an overall risk ratio (RR) for symptomatic UTIs treated with AP versus placebo of 0.38. Applied to an expected UTI rate under 3-day AP of 9%, this corresponds to an expected (hypothetical) UTI rate under placebo of 23.68% (i.e. 0.2368 * 0.38 = 0.09) and an expected absolute treatment effect (absolute risk reduction, ARR) of 3-day AP versus placebo (M1) of 14.68% (ARR = 0.2368 – 0.09 = 0.1468). The non-inferiority margin was determined in order to preserve 70% of M1 (i.e. ARR*0.7; 10.28% on an absolute scale), which was judged a clinically important fraction. This would correspond to an absolute event rate under 1-day AP versus placebo of 13.4% (i.e. 0.2368 – 0.1028 = 0.134). Assuming an event rate of 0.09 (9 %) in both trial arms, this resulted in a non-inferiority margin $\delta$ = 4.4% (i.e. 13.4% - 9%).

In other words, when 100 patients undergoing TURP or GL would receive no treatment, 24 (i.e. 23.68%) would have a symptomatic UTI and 76 patients not. Would all these 100 be treated with 3 day AP, 9 (i.e. 9%) would still have a symptomatic UTI and 91 not. Would all these 100 be treated with a single AP (which still has 70% of the effect), approximately 13 patients would have a symptomatic UTI and 87 not.

We would consider it acceptable when approximately 4 more of 100 patients would have an UTI when bearing in mind that these 4 UTIs can be treated relatively straightforward with antibiotics and that simultaneously the use of AP can be strongly decreased in all 100 treated patients. This also very likely leads to fewer individual adverse events and resistant pathogens, and beyond this has important beneficial consequences for public heath due to lesser antibiotic resistance in general.

The sample size was calculated using a re-sampling procedure. Each sample size, n_i_=1,...,111 = 800, ..., 3000, was evaluated by simulating 999 individual samples of n_i_ patients based on the assumptions described above. The difference in the observed event rates p_idAP_ – p_3dAP_ was compared with the non-inferiority margin δ, using a two-sided 95 % confidence interval, calculated using a continuity-corrected modification of Wilson’s score method (41). Non-inferiority was declared if the upper limit of this 95 % confidence interval was no larger than the non-inferiority margin δ. Figure 2 shows the relationship between the total sample size (number of patients, not including drop-outs) and the expected absolute difference in the UTI rate between the two trial arms. For an assumed event rate of 9% in both trial arms, and a δ of 4.4%, a total of 1574 patients should be recruited, in order to have 1416 evaluable patients.


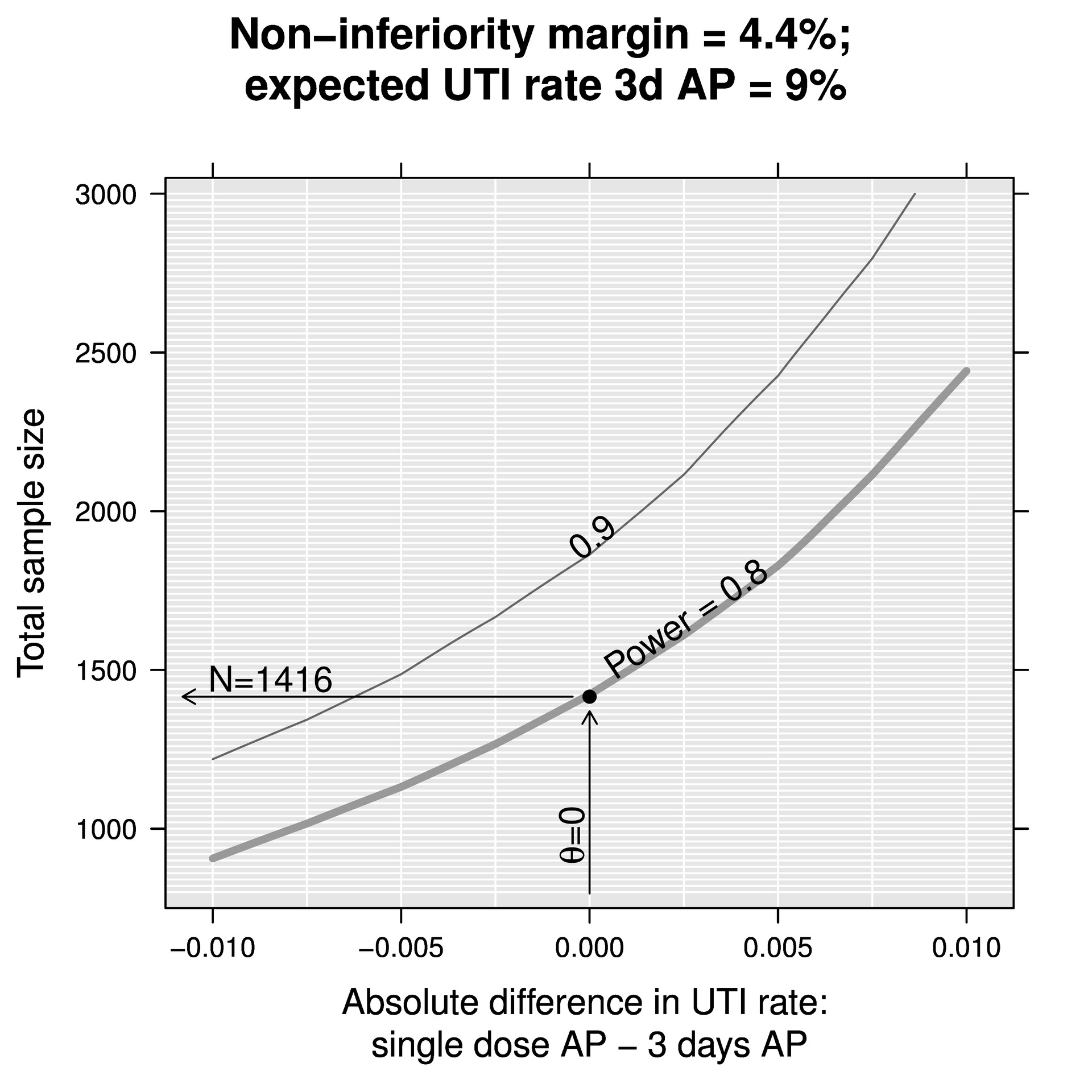
**Figure 2:** Total sample size needed to show possible non-inferiority of single-dose AP to 3-day AP, depending on the absolute difference in the UTI rate. An examples is shown for an absolute difference of 0% and a power of 80%. The curves are smoothed and for illustration only.

## Statistical criteria of termination of trial

Not applicable.

## Planned Analyses

### 11.4.1 Datasets to be analysed, analysis populations

Patients will be analysed according to the intention to treat (ITT) principle, i.e. all patients will be analysed according to the treatment group they were allocated to. Missing values will be handled as described in Section 11.5.

The per protocol (PP) analysis will include all patients without any major protocol deviation and for which the endpoint data is available (i.e. complete case analysis). Patients who miss more than one oral dose of study medication or who did not receive the intravenous TMP/SMX due to any reasons, will be classified as non-adherent to study drug and will be excluded from the per-protocol analysis.

### 11.4.2 Primary Analysis

As primary analysis, the difference in the proportion in UTIs between the single-dose AP and the 3-day AP arm, will be compared with the non-inferiority margin using a two-sided 95% confidence interval calculated according to the continuity-corrected modification of Wilson’s score method (41).

Problems arising from misclassification of outcomes or from high attrition (missing data) that may “dilute” possible differences between groups are of special importance in non-inferiority trials. This may lead to falsely claiming non-inferiority in ITT analyses. However, per protocol analyses require strong assumptions and are prone to time-varying confounding (which may require careful adjustment for post-randomisation occurrences, for example treatment switches that would represent a protocol violation). However, post-randomisation confounding does not affect the causal interpretation of the ITT analyses. There is no consensus as to the choice of the primary analysis in non-inferiority trials (42).

This is a pragmatic trial aiming to inform the practical question about how to act, i.e. we aim to compare two treatment strategies. Therefore we will use the ITT analysis as our main analysis since it is the only method to estimate the unbiased causal effect of the intention to use the two treatment strategies (i.e. the intention to begin and adhere to a 1 vs 3 day AP). However, this has the risk that the different explanatory question about the causal effect of actually using the antibiotic treatment as specified in the protocol (i.e. under the -unrealistic- assumption of perfect adherence in all patients) will be more or less biased. This explanatory question, however, can only be evaluated in a per protocol analysis under strong and unrealistic assumptions (i.e. that all post-randomisation confounder leading to non-adherence are known, measured, and well understood so that they can be accurately modelled). Within the framework of this trial it is impossible to measure all possible post-randomisation confounders (e.g. patients’ preferences and values which may lead to non-adherence or treatment switch). Nevertheless, acknowledging these issues, we will use a PP analysis to estimate this per protocol effect. We assume that in this clinical situation only very few patients will be “non-adherent” due to the very short treatment period and that missing data is rarely (if ever) associated with the treatment allocation (i.e. missing completely at random) leading to small differences between the two estimates. In our publications (and other study reports), we will report the results of both analyses, discuss any differences and carefully describe the conceptual differences of both analyses

**Sensitivity analysis:** In order to evaluate the robustness of the imputation method the ITT analysis of the primary analysis will be repeated using inverse probability censored weighting (IPCW) methodology (details see Section 11.5).

**Subgroup analysis:** The difference in the UTI rate between the study arms will be estimated and reported with 95% confidence interval separately for patients treated with TURP and patients treated with GL. No other subgroups are pre-specified.

### 11.4.3 Secondary Analyses

Secondary analyses will be exploratory in nature and aim to measure the ITT effect. All estimates will be presented with 95% confidence intervals.

The key secondary outcome a (Symptomatic UTI (based on clinical diagnosis supported by measured bacteriuria of ≥10^5^ cfu/ml) treated with antibiotics*)* will be analysed as described for the primary endpoint (Section 11.4.2). Results (size and direction of the effect) will be compared informally to the primary endpoint.

Secondary outcomes *b – m* (see Section 5.2) will be analysed with *generalised linear mixed models (GLMM) with binomial error distribution*, including trial arm as predictor and study centre as random effect. Estimates will be reported as odds ratios (OR).

Secondary outcomes *n – p* (see Section 5.2) will be treated as count data and analysed with *GLMM with poisson error distribution*, including trial arm as predictor and study centre as random effect. Estimates will be reported as relative effects.

Secondary outcomes *r – s* (see Section 5.2) will be analysed by means of linear mixed models (LMM), including trial arm as predictor and study centre as random effect. Estimates will be reported as absolute effects.

### 11.4.4 Interim analyses

No interim analyses will be done.

### 11.4.5 Safety analysis

Safety endpoints (Section 10.1.1) will be reported for each study arm using summary statistics (frequencies and percentages). No statistical tests are planned for the safety endpoints.

### 11.4.6 Deviation(s) from the original statistical plan

If substantial deviations of the analysis as outlined in these sections are needed for whatever reason, the protocol will be amended. All deviations of the analysis from the protocol or from the detailed analysis plan will be listed and justified in a separate section of the final statistical report.

## Handling of missing data and drop-outs

Drop-outs will not be replaced. The number of drop-outs will be reported for each trial arm separately, indicating the reason for drop-out.

**Primary analysis:** For the ITT analysis, missing values will be imputed using multiple imputation methods. As sensitivity analysis, the analysis will be repeated using inverse probability censored weighting (IPCW) methodology (43). This approach accounts for missingness based on a logistic regression model for the probability of having a complete follow-up given past history covariates and outcomes. By using inverse weights, the impact of underrepresented subjects is inflated, so that one can observe associations that would have been observed if all subjects had stayed in the study, assuming the model is correctly specified. For the PP analysis, a complete case approach is applied where no missing values are possible.

**Secondary analyses:** Secondary analyses will be performed primarily as complete case analyses. For the key secondary endpoint [a] and secondary endpoints [r] and [s], additional (sensitivity) analyses will be performed, as described for the primary endpoint. For all other secondary endpoints, missing data will only be imputed or IPCW applied if the amount of missing values is considerable (i.e. more than 5% of values missing in one treatment group, more missing values than events in one group). This means that the number of patients may vary among analyses.

# QUALITY ASSURANCE AND CONTROL

## Data handling and record keeping / archiving

### Case Report Forms

Study data will be recorded in an electronic CRFs (eCRF). A case report form (CRF) will also be available at the study sites if personnel prefers to collect data in a first step on a printed form. Since the vast majority of data will be collected by a study nurse from the hospital information system, anonymisation of the CRF and eCRF would not result in any additional patient protection. However, only local investigators will be able to see names and birthdays of patients from their study site within the eCRF.

### 12.1.2 Specification of source documents

Source data must be available at the study site to document the existence of the study participants. Source data must include the original documents related to the study, as well as the medical treatment and medical history of the participant.

### 12.1.3 Record keeping / archiving

The study eCRF will be locked after all data was entered or transferred. The complete dataset is exported and sent to the Sponsor/Principle Investigator via a secured channel and according to Standard operation procedure (SOPs) of the CTU Basel.

All study data will be archived for a minimum of 10 years after study termination or premature termination of the trial.

## Data management

### 12.2.1 Data Management System

The clinical trial data will be collected in an EDC system named secuTrial^®^. The EDC system runs on a server maintained by the IT department of the University Hospital Basel. The eCRF is implemented by a data manager at the CTU at the University Hospital Basel. All data mentioned under 9.1 are collected by the study nurse during study phase. Study nurses will enter the data into the database based on those like prescribed under 9.2.1 and 9.2.2.

### 12.2.2 Data security, access and back-up

The EDC system is accessible via a standard browser on a WWW-connected device. Password protection ensures that only authorised persons can enter the system to view, add or edit data according to their permissions. Access to the data will be physically limited to study personnel and only data managers and biostatisticians of the study will be given access codes to the data.

User administration is done by the CTU Basel according to their processes. All personal inserting and editing data must be trained by the CTU Basel or by already trained staff of the study. A regular backup of the data base is done according to the processes of the IT department of the University Hospital Basel.

### 12.2.3 Analysis and archiving

At the end of the study all raw data, processing algorithms and analyses code will be transferred in duplicate to optical storage mediums (DVDs) and will be securely archived at the Department of Urology, University Hospital Basel.

### 12.2.4 Electronic and central data validation

Data entered into the eCRF will be validated for completeness and discrepancies automatically. An audit trail system maintains a record of initial entries and changes (reasons, time and date of changes, user identification of entry and changes).

## Monitoring

The Sponsor/Principle Investigator and a designated study monitor from CTU Basel will conduct a site initiation visit at each study site to verify the qualifications of the local investigators, inspect the site facilities, and inform the investigators of responsibilities and the procedures for ensuring adequate and correct documentation and use of the EDC system. In addition, the study monitor from the CTU Basel will conduct two routine monitoring visits per site, the first after inclusion of 1-2 participants, the second after inclusion of the last participant, as well a site closure visit at the end of the study to resolve any remaining queries. The purpose of the visits is to confirm the following: The study is being conducted according to the protocol and within the specified time frame. The data are being collected accurately and completely on the eCRFs and source documents. The study medication is being correctly administered. Adverse events are being correctly reported. The facilities and staff remain adequate. The local investigators ensure that source data and documents are made accessible to the study monitor and answer questions by the study monitor. Details for the monitoring visits will be described in the final Monitoring Plan, which will be agreed by the Sponsor and the monitor of the CTU Basel.

## Audits and Inspections

The study documentation and the source data/documents are accessible to auditors/inspectors and questions are answered during inspections. All involved parties must keep the patient data strictly confidential.

## Confidentiality, Data Protection

Data access to source documents will be permitted for purposes of monitoring, audits and inspections. Access to data is described in Section 12.2.2.

## Storage of biological material and related health data

No biological samples will be collected in the frame of the CITrUS study (i.e. only data from routinely collected samples will be used).

# PUBLICATION AND DISSEMINATION POLICY

All trials results will be published with open access in peer-reviewed journal publications. We intend to publish the study protocol of the CITrUS Trial in a peer reviewed journal. Authorship to publications will be granted according to the rules of the International Committee of Medical Journal Editors (ICMJE).

# FUNDING AND SUPPORT

## Funding

This study is funded by a grant from the Swiss National Science Foundation (NFP 72 – Grant No SNF407240_177492; <http://www.nfp72.ch/en/projects/module-3-optimised-use-of-antibiotics/detecting-excessive-antibiotic-use-in-urology>).

## Other Support

Not applicable.

# INSURANCE

Insurance will be provided by the Sponsor. The insurance certificate will be acquired after ethical approval is provided. Proof of the insurance certificate will be submitted to the local ethics committees before the enrolment of the first patient. A copy of the certificate is filed in each investigator site file and the trial master file.

# REFERENCES

1. Reich O, Gratzke C, Bachmann A, Seitz M, Schlenker B, Hermanek P, et al. Morbidity, mortality and early outcome of transurethral resection of the prostate: a prospective multicenter evaluation of 10,654 patients. J Urol. 2008;180(1):246-9.

2. Ruszat R, Wyler S, Forster T, Reich O, Stief CG, Gasser TC, et al. Safety and effectiveness of photoselective vaporization of the prostate (PVP) in patients on ongoing oral anticoagulation. Eur Urol. 2007;51(4):1031-8; discussion 8-41.

3. Allegranzi B, Bischoff P, de Jonge S, Kubilay NZ, Zayed B, Gomes SM, et al. New WHO recommendations on preoperative measures for surgical site infection prevention: an evidence-based global perspective. The Lancet Infectious Diseases.16(12):e276-e87.

4. Bonkat G, Pickard R, Bartoletti R, et al. EAU Guidelines on Urological Infections. Perioperative antibacterial prophylaxis in urology. In: *Complete European Association of Urology Guidelines. Edn. presented at the EAU Annual Congress London 2017;* 2017: 35-9.

5. Alsaywid BS, Smith GH. Antibiotic prophylaxis for transurethral urological surgeries: Systematic review. Urol Ann. 2013;5(2):61-74.

6. Qiang W, Jianchen W, MacDonald R, Monga M, Wilt TJ. Antibiotic prophylaxis for transurethral prostatic resection in men with preoperative urine containing less than 100,000 bacteria per ml: a systematic review. J Urol. 2005;173(4):1175-81.

7. Berry A, Barratt A. Prophylactic antibiotic use in transurethral prostatic resection: a meta-analysis. J Urol. 2002;167(2 Pt 1):571-7.

8. Wagenlehner FM, Wagenlehner C, Schinzel S, Naber KG, Working Group "Urological Infections" of German Society of U. Prospective, randomized, multicentric, open, comparative study on the efficacy of a prophylactic single dose of 500 mg levofloxacin versus 1920 mg trimethoprim/sulfamethoxazole versus a control group in patients undergoing TUR of the prostate. Eur Urol. 2005;47(4):549-56.

9. Bachmann A, Ruszat R, Wyler S, Reich O, Seifert HH, Muller A, et al. Photoselective vaporization of the prostate: the basel experience after 108 procedures. Eur Urol. 2005;47(6):798-804.

10. Bachmann A, Schurch L, Ruszat R, Wyler SF, Seifert HH, Muller A, et al. Photoselective vaporization (PVP) versus transurethral resection of the prostate (TURP): a prospective bi-centre study of perioperative morbidity and early functional outcome. Eur Urol. 2005;48(6):965-71; discussion 72.

11. Bachmann A, Tubaro A, Barber N, d'Ancona F, Muir G, Witzsch U, et al. A European multicenter randomized noninferiority trial comparing 180 W GreenLight XPS laser vaporization and transurethral resection of the prostate for the treatment of benign prostatic obstruction: 12-month results of the GOLIATH study. J Urol. 2015;193(2):570-8.

12. Hueber PA, Liberman D, Ben-Zvi T, Woo H, Hai MA, Te AE, et al. 180 W vs 120 W lithium triborate photoselective vaporization of the prostate for benign prostatic hyperplasia: a global, multicenter comparative analysis of perioperative treatment parameters. Urology. 2013;82(5):1108-13.

13. Battegay M. BV, Bloch-Infanger C. . Infektiologie und Spitalhygiene, Weissbuch 2016. 2016:233.

14. Cek M, Tandogdu Z, Naber K, Tenke P, Wagenlehner F, van Oostrum E, et al. Antibiotic prophylaxis in urology departments, 2005-2010. Eur Urol. 2013;63(2):386-94.

15. Bausch K, Roth JA, Seifert HH, Widmer AF. Overuse of antimicrobial prophylaxis in low-risk patients undergoing transurethral resection of the prostate. Swiss Med Wkly. 2018;148:w14594.

16. Woodford N, Ward ME, Kaufmann ME, Turton J, Fagan EJ, James D, et al. Community and hospital spread of Escherichia coli producing CTX-M extended-spectrum beta-lactamases in the UK. J Antimicrob Chemother. 2004;54(4):735-43.

17. Tacconelli E, Magrini N. GLOBAL PRIORITY LIST OF ANTIBIOTIC-RESISTANT BACTERIA TO GUIDE RESEARCH, DISCOVERY, AND DEVELOPMENT OF NEW ANTIBIOTICS. World Health Organisation. 2017.

18. Bonkat G, Muller G, Braissant O, Frei R, Tschudin-Suter S, Rieken M, et al. Increasing prevalence of ciprofloxacin resistance in extended-spectrum-beta-lactamase-producing Escherichia coli urinary isolates. World J Urol. 2013;31(6):1427-32.

19. Blaettler L, Mertz D, Frei R, Elzi L, Widmer AF, Battegay M, et al. Secular trend and risk factors for antimicrobial resistance in Escherichia coli isolates in Switzerland 1997-2007. Infection. 2009;37(6):534-9.

20. Bantar C, Sartori B, Vesco E, Heft C, Saul M, Salamone F, et al. A hospitalwide intervention program to optimize the quality of antibiotic use: impact on prescribing practice, antibiotic consumption, cost savings, and bacterial resistance. Clin Infect Dis. 2003;37(2):180-6.

21. Cai T, Verze P, Brugnolli A, Tiscione D, Luciani LG, Eccher C, et al. Adherence to European Association of Urology Guidelines on Prophylactic Antibiotics: An Important Step in Antimicrobial Stewardship. Eur Urol. 2016;69(2):276-83.

22. Hargreave TB, Botto H, Rikken GH, Hindmarsh JR, McDermott TE, Mjolnerod OK, et al. European collaborative study of antibiotic prophylaxis for transurethral resection of the prostate. Eur Urol. 1993;23(4):437-43.

23. Hargreave TB, Gould JC, Kinninmonth AW, Jeffrey RR, Varma JS, Macintyre CC, et al. A randomized trial of 48 hours of prophylactic cefotaxime versus single dose in transurethral prostatic surgery. J Antimicrob Chemother. 1984;14 Suppl B:263-9.

24. Tsugawa M, Hashimoto H, Monden K, Kumon H, Ohmori H. [Antimicrobial prophylaxis in transurethral resection of the prostate]. Nihon Hinyokika Gakkai Zasshi. 1998;89(4):453-9.

25. Hall JC, Christiansen KJ, England P, Low AI, McRae PJ, Mander J, et al. Antibiotic prophylaxis for patients undergoing transurethral resection of the prostate. Urology. 1996;47(6):852-6.

26. Costa FJ. Lomefloxacin prophylaxis in visual laser ablation of the prostate. Urology. 1994;44(6):933-6.

27. Thorsteinsson SB, Einarsson GV, Steingrimsson O, Jensen RH. Antibiotic prophylaxis in transurethral surgery. Ceftriaxone versus cephradine versus control. A randomized, prospective study in low risk patients. Scand J Infect Dis Suppl. 1990;70:68-73.

28. Duclos JM, Larrouturou P, Sarkis P. Timing of antibiotic prophylaxis with cefotaxime for prostatic resection: better in the operative period or at urethral catheter removal? Am J Surg. 1992;164(4A Suppl):21S-3S.

29. Iversen P, Madsen PO. Short-term cephalosporin prophylaxis in transurethral surgery. Clin Ther. 1982;5 Suppl A:58-66.

30. Bouassida K, Jaidane M, Bouallegue O, Tlili G, Naija H, Mosbah AT. Nosocomial urinary tract infections caused by extended-spectrum beta-lactamase uropathogens: Prevalence, pathogens, risk factors, and strategies for infection control. Can Urol Assoc J. 2016;10(3-4):E87-93.

31. Schneidewind L, Kranz J, Schlager D, Barski D, Muhlsteadt S, Grabbert M, et al. Mulitcenter study on antibiotic prophylaxis, infectious complications and risk assessment in TUR-P. Cent European J Urol. 2017;70(1):112-7.

32. Centers for Disease Control and Prevention (CDC). Urinary Tract Infection (Catheter-Associated Urinary Tract Infection [CAUTI] and Non-Catheter-Associated Urinary Tract Infection [UTI]) and Other Urinary System Infection [USI]) Events. <https://www.cdc.gov/nhsn/PDFs/pscManual/7pscCAUTIcurrent.pdf> [accessed 12 June 2018].

33. WHO Collaborating Centre for Drug Statistics Methodology. ATC/DDD Index 2018. <https://www.whocc.no/atc_ddd_index/> [accessed: 11 May 2018].

34. Hygienemaßnahmen bei Infektionen oder Besiedlung mit multiresistenten gramnegativen Stäbchen. Empfehlung der Kommission für Krankenhaushygiene und Infektionsprävention (KRINKO) beim Robert Koch-Institut (RKI). Bundesgesundheitsbl 2012. 55:1311–1354. DOI 10.1007/s00103-012-1549-5.

35. Crobach MJ, Planche T, Eckert C, Barbut F, Terveer EM, Dekkers OM, et al. European Society of Clinical Microbiology and Infectious Diseases: update of the diagnostic guidance document for Clostridium difficile infection. Clin Microbiol Infect. 2016;22 Suppl 4:S63-81.

36. Internationaler Prostata Symptomen-Score (IPSS). <http://www.dr-walser.ch/ipss.pdf> [accessed: 21 July 2018].

37. Wadie BS, Ibrahim EH, de la Rosette JJ, Gomhha MA, Ghoneim MA. The relationship of the International Prostate Symptom Score and objective parameters for diagnosing bladder outlet obstruction. Part I: when statistics fail. J Urol; 165(1):32-4.

38. ICH Harmonised Tripartite Guideline. Clinical Safety Data Management: Definitions and Standards for Expected Reporting E2A. <http://academy.gmp-compliance.org/guidemgr/files/E2A_GUIDELINE.PDF> [accessed: 24 May 2018]

39. Scholz M, Luftenegger W, Harmuth H, Wolf D, Holtl W. Single-dose antibiotic prophylaxis in transurethral resection of the prostate: a prospective randomized trial. Br J Urol. 1998;81(6):827-9.

40. U.S. Department of Health and Human Services (National Institutes of Health; National Cancer Institute). Common Terminology Criteria for Adverse Events (CTCAE). Version 5.0. Published: November 27, 2017. <https://ctep.cancer.gov/protocoldevelopment/electronic_applications/docs/CTCAE_v5_Quick_Reference_8.5x11.pdf> [accessed: 15 May 2018].

41. Newcombe RG. Interval estimation for the difference between independent proportions: comparison of eleven methods. Stat Med. 1998;17(8):873-90.

42. Rehal S, Morris TP, Fielding K, Carpenter JR, Phillips PP. Non-inferiority trials: are they inferior? A systematic review of reporting in major medical journals. BMJ Open. 2016;6(10):e012594.

43. Robins JM, Hernan MA, Brumback B. Marginal structural models and causal inference in epidemiology. Epidemiology. 2000;11(5):550-60.

# APPENDICES

## Search strategy

("anti-bacterial agents"[Pharmacological Action] OR "anti-bacterial agents"[MeSH Terms] OR "antibiotic"[tiab] OR "antibiotics"[tiab] OR anti-bacterial agents[tiab] OR anti-bacterial agent[tiab] OR antibacterial agents[tiab] OR antibacterial agent[tiab] OR Bacteriocidal Agents[tiab] OR Bacteriocidal Agent[tiab] OR Bacteriocides[tiab] OR Bacteriocide[tiab]) AND (((Transurethral[tiab] OR greenlight[tiab] OR green light[tiab] OR laser[tiab] OR Photoselective vaporisation[tiab] OR Photoselective vaporization[tiab]) AND (prostate[tiab] OR "Prostate"[Mesh] OR Prostatectomy[tiab] OR "Prostatectomy"[Mesh] OR Prostatectomies[tiab])) OR "transurethral resection of prostate"[MeSH Terms] OR TURP[tiab]) AND (randomized controlled trial[pt] OR controlled clinical trial[pt] OR randomized[tiab] OR placebo[tiab] OR "clinical trials as topic"[MeSH Terms:noexp] OR randomly[tiab] OR trial[ti] NOT ("animals"[MeSH Terms] NOT "humans"[MeSH Terms]))

Searched: 20. March 2018, PubMed; 117 Hits
