# Supplementary material for: Single-dose versus 3-day cotrimoxazole prophylaxis in transurethral resection or greenlight laser vaporisation of the prostate: study protocol for a multicentre randomised placebo controlled non-inferiority trial (CITrUS trial)
Source: Trials. 2019 Feb 19;20:142. doi: 10.1186/s13063-019-3237-3 (PMC6381623; doi:10.1186/s13063-019-3237-3)
Supplement: Supplementary file 2 — World Health Organization Trial Registration Data Set. (DOCX 23 kb) [file 13063_2019_3237_MOESM2_ESM.docx]

**Single-Dose Versus 3-Day Cotrimoxazole Prophylaxis in Transurethral Resection or Greenlight Laser Vaporisation of the Prostate: Study Protocol for a Multicentre Randomised Placebo Controlled Non-Inferiority Trial (CITrUS trial)**

Benjamin Speich, Kathrin Bausch, Jan A. Roth, Lars G. Hemkens, Hannah Ewald, Deborah R. Vogt, Nicole Bruni, Stephanie Deuster, Hans-H. Seifert, Andreas F. Widmer

#### WHO Trial Registration Data Set (Version 1.3.1)

#### Statement was filled out on the 22. October 2018.

1. **Primary Registry and Trial Identifying Number**

Clinicaltrials.gov. NCT03633643

1. **Date of Registration in Primary Registry**

16. August 2018

1. **Secondary Identifying Numbers**

Not applicable

1. **Source(s) of Monetary or Material Support**

This study is funded by a grant from the Swiss National Science Foundation (NFP 72 – Grant No SNF407240_177492). The funding source had no role in the design of the study and will not have any role during its execution, analyses, interpretation of the data, nor in the decision for publication of the results.

1. **Primary Sponsor**

| Sponsor:  Principal Investigator/Sponsor Investigator: | University Hospital Basel  Prof. Dr. med. Widmer, Andreas F.  Division of Infectious Diseases and Hospital Epidemiology  University Hospital Basel  Petersgraben 4  4031 Basel  Switzerland  Phone: +41 61 265 38 51  E-Mail: [andreas.widmer@usb.ch](mailto:andreas.widmer@usb.ch) |
| --- | --- |

1. **Secondary Sponsor(s)**

**Not applicable**

1. **Contact for Public Queries**

Dr. Kathrin Bausch

Department of Urology

University Hospital Basel

Spitalstrasse 21

4056 Basel

Switzerland

Phone: + 41 61 556 51 97

E-Mail: kathrin.bausch@usb.ch

1. **Contact for Scientific Queries**

| Sponsor:  Principal Investigator/Sponsor Investigator: | University Hospital Basel  Prof. Dr. med. Widmer, Andreas F.  Division of Infectious Diseases and Hospital Epidemiology  University Hospital Basel  Petersgraben 4  4031 Basel  Switzerland  Phone: +41 61 265 38 51  E-Mail: [andreas.widmer@usb.ch](mailto:andreas.widmer@usb.ch) |
| --- | --- |

1. **Public Title**

Antibiotische Prophylaxe in der Urologie – CITrUS

1. **Scientific Title**

Title: Single-Dose Versus 3-Day Cotrimoxazole Prophylaxis in Transurethral Resection or Greenlight Laser Vaporisation of the Prostate: A Pragmatic, Multicentre Randomised Placebo Controlled Non-Inferiority Trial

Short title: Cotrimoxazole Prophylaxis in Transurethral Resection or Greenlight Laser Vaporisation of the Prostate

Study identifier: CITrUS (single-dose versus 3-day CotrImoxazole prophylaxis in TransUrethral resection of the prostate, Switzerland)

1. **Countries of Recruitment**

Switzerland

1. **Health Condition(s) or Problem(s) Studied**

Male with voiding disorder

1. **Intervention(s)**

Group A

Single-dose TMP/SMX (i.e. Cotrimoxazole) perioperative as two ampoules of TMP/SMX 400/80 mg (Bactrim Inf Konz^®^) solved in 250 ml sodium chloride short infusion followed by five oral applications of placebo (lactose tablet; Fagron GmbH & Co. KG) at the evening of the surgery and thereafter twice daily on day 1 and 2 after surgery while the patient is in hospital.

Group B

3-day AP with TMP/SMX (i.e. Cotrimoxazole): Preoperatively as two ampoules of TMP/SMX 400/80mg (Bactrim Inf Konz®) solved in 250 ml sodium chloride short infusion, followed by five oral applications of TMP/SMX 800/160 mg (Nopil forte^®^ tablets) at the evening of the surgery and thereafter twice daily on day 1 and 2 after surgery while the patient is in hospital.

1. **Key Inclusion and Exclusion Criteria**

#### Inclusion criteria

- Adult male patients (≥18 years)
- Obstructive voiding disorder (e.g. benign prostate hyperplasia, obstructive prostate cancer)
- Planned TURP or GL

#### Exclusion criteria

- Evidence for (catheter associated-) UTI, with or without antibiotic treatment in the last 7 days prior to randomisation.
- Any evidence of a history of positive urine culture (cfu ≥10^5^/ml in midstream-urine with no more than two species) and resistance to TMP/SMX in the last 7 days prior to randomisation.
- Known contraindication against study drugs according to the Swissmedic package leaflet (e.g. known liver dysfunction, renal insufficiency; patients with glomerular filtration rate (calculated by the MDRD or CKD-EPI) <30ml/min or dialysis patients will be excluded).
- Antibiotic treatment for any reason within 7 days prior to randomisation
- Indication for AP for other reasons (e.g. endocarditis prophylaxis, transplanted patients under systemic immunosuppression).

1. **Study Type**

This is a randomised controlled, non-inferiority, parallel group, trial with blinded treating physicians, patients and outcome assessors using a 1:1 randomisation ratio in five urological departments in Switzerland.

1. **Date of First Enrollment**

Anticipated November 2018

1. **Sample Size**

Planned: 1574

1. **Recruitment Status**

Pending: participants are not yet being recruited or enrolled at any site

1. **Primary Outcome(s)**

Symptomatic UTI (based on clinical diagnosis) treated with antimicrobial agents.

All outcomes are events within 30 days (±5 days) after randomisation if not stated otherwise.

1. **Key Secondary Outcomes**

#### Symptomatic UTI (based on clinical diagnosis supported by measured bacteriuria of ≥10^5^ cfu/ml) treated with antimicrobial agents (key secondary outcome)

- Symptomatic cystitis (based on clinical diagnosis)
- Symptomatic epididymitis (based on clinical diagnosis)
- Symptomatic pyelonephritis (based on clinical diagnosis)
- Symptomatic prostatitis (based on clinical diagnosis)
- Symptomatic urethritis (based on clinical diagnosis)
- Urosepsis (based on clinical diagnosis)
- Prescription of antibiotics (for any reason)
- Prescribed defined daily doses (DDD) of antibiotics (cumulative sum of DDD from randomisation to day 30)
- Asymptomatic bacteriuria of ≥10^5^ cfu/ml treated with antimicrobial agents
- Detection of multidrug-resistant bacteria in urine culture (3MRGN, 4MRGN)
- Any *Clostridium difficile*-associated infection
- Duration of catheterisation (cumulative sum of days between randomisation and end of catheterisation or day 30)
- Duration of hospital stay (cumulative sum of hospital days between randomisation and day 30)
- Duration of intensive care unit stay (cumulative sum of ICU days between randomisation and day 30)
- Re-hospitalisation (within 30 days after randomisation)
- Change of International Prostate Symptom Score (prior to randomisation and at day 30 after randomisation)
- Change of Quality of life Score (prior to randomisation and at day 30 after randomisation)
- All-cause mortality
- Total adverse events
- Total serious adverse events

All outcomes are events within 30 days (±5 days) after randomisation if not stated otherwise.

1. **Ethics Review**

Ethical approval has been obtained from the ethical committees ‘Nordwest- und Zentralschweiz’ and Zürich (reference number 2018-01404). Additionally approval from Swissmedic was obtained (reference number 2018DR4111).

1. **Completion date**

Not applicable

1. **Summary Results**

Not applicable

1. **IPD sharing statement**

Individual patient data will be shared on reasonable request by contacting the principle investigator.
